# Interacting Bioenergetic and Stoichiometric Controls on Microbial Growth

**DOI:** 10.3389/fmicb.2022.859063

**Published:** 2022-05-17

**Authors:** Arjun Chakrawal, Salvatore Calabrese, Anke M. Herrmann, Stefano Manzoni

**Affiliations:** ^1^Department of Physical Geography, Stockholm University, Stockholm, Sweden; ^2^Bolin Centre for Climate Research, Stockholm University, Stockholm, Sweden; ^3^Department of Biological and Agricultural Engineering, Texas A&M University, College Station, TX, United States; ^4^Department of Soil and Environment, Swedish University of Agricultural Sciences, Uppsala, Sweden

**Keywords:** microbial growth, nitrogen limitation, energy limitation, thermodynamics, bioenergetics, stoichiometry, DNRA, denitrification

## Abstract

Microorganisms function as open systems that exchange matter and energy with their surrounding environment. Even though mass (carbon and nutrients) and energy exchanges are tightly linked, there is a lack of integrated approaches that combine these fluxes and explore how they jointly impact microbial growth. Such links are essential to predicting how the growth rate of microorganisms varies, especially when the stoichiometry of carbon- (C) and nitrogen (N)-uptake is not balanced. Here, we present a theoretical framework to quantify the microbial growth rate for conditions of C-, N-, and energy-(co-) limitations. We use this framework to show how the C:N ratio and the degree of reduction of the organic matter (OM), which is also the electron donor, availability of electron acceptors (EAs), and the different sources of N together control the microbial growth rate under C, nutrient, and energy-limited conditions. We show that the growth rate peaks at intermediate values of the degree of reduction of OM under oxic and C-limited conditions, but not under N-limited conditions. Under oxic conditions and with N-poor OM, the growth rate is higher when the inorganic N (N_Inorg_)-source is ammonium compared to nitrate due to the additional energetic cost involved in nitrate reduction. Under anoxic conditions, when nitrate is both EA and N_Inorg_-source, the growth rates of denitrifiers and microbes performing the dissimilatory nitrate reduction to ammonia (DNRA) are determined by both OM degree of reduction and nitrate-availability. Consistent with the data, DNRA is predicted to foster growth under extreme nitrate-limitation and with a reduced OM, whereas denitrifiers are favored as nitrate becomes more available and in the presence of oxidized OM. Furthermore, the growth rate is reduced when catabolism is coupled to low energy yielding EAs (e.g., sulfate) because of the low carbon use efficiency (CUE). However, the low CUE also decreases the nutrient demand for growth, thereby reducing N-limitation. We conclude that bioenergetics provides a useful conceptual framework for explaining growth rates under different metabolisms and multiple resource-limitations.

## Introduction

Microorganisms (chemoheterotrophs) depend on organic matter (OM) not only as a carbon (C) source but also as an energy source. From a bioenergetics perspective, a single microbial cell can be considered as a system or an “engine” that converts dead OM into living cells. This engine also generates the energy required *via* an exchange of electrons with the surrounding environment (Roels, 1980a; Kleerebezem and Van Loosdrecht, 2010; Stockar, 2010). Specifically, during growth, microorganisms catabolize OM that generates the electrons (therefore, OM is an electron donor) taken up by terminal electron acceptors (EAs), thereby creating a redox system driven by the changes in Gibbs energy. A fraction of this energy is then utilized by microbial cells for maintaining and producing more cells (anabolism), while the rest is dissipated into the environment (McCarty, 2007; Smeaton and Van Cappellen, 2018; Calabrese et al., 2021). This balance of electrons from the catabolic and anabolic reactions in the form of Gibbs energies frames the bioenergetic theory of microbial growth (Von Stockar et al., 2006; Amend and LaRowe, 2019).

In microbial ecology, energy- and C-limitations are often confounded (Burgin et al., 2011). However, based on the bioenergetic theory of microbial growth, these two controlling factors can be independent, due to the different degree of reduction of the OM (or the energy content of OM), defined as the number of electrons produced per C-mol of OM in a complete oxidation reaction. For example, when C is not limiting, microorganisms growing on glucose would have a higher growth rate compared to oxalate because of the higher number of electrons produced per C-mol of glucose catabolized, which results in higher carbon use efficiency (CUE) (the ratio of growth over C-uptake) (Roels, 1980a; Heijnen et al., 1992). Moreover, how much energy can be extracted from catabolism also depends on which EAs are available. For example, when C is not limiting, the microorganisms that are catabolizing glucose under oxic conditions with oxygen as the EA would have a higher growth rate compared to those under anoxic conditions with iron as the EA (LaRowe and Amend, 2015). Energy-limitation not only reduces microbial growth by decreasing CUE (Roels, 1980a; Heijnen et al., 1992; Bölscher et al., 2016; Calabrese et al., 2021) but also alters microbial physiology (e.g., dormancy) under an extremely low substrate-availability (Lever et al., 2015). We consider a microbial system as energy-limited when the energy content of OM or the availability of thermodynamically preferred EA constrains growth (Lever et al., 2015), whereas it is C-limited when the C content of OM constrains growth. The combinations of C and energy availabilities create a spectrum of conditions ranging from relatively more C- to more energy-limited and (often) with co-limitation of these two resources.

The importance of energy-limitations on OM degradation is often studied in anoxic environments (e.g., marine sediments, groundwater) because of the low availability of high energy yielding EAs (Hoehler and Jørgensen, 2013; Lever et al., 2015; Bradley et al., 2020; LaRowe et al., 2020). However, OM decomposition can also be inhibited by EA or degree of reduction of OM under fluctuating oxic and anoxic conditions found in paddy fields (Fan et al., 2020; Li et al., 2021), wetlands and humid tropical forest soils (Hall et al., 2013; Wang et al., 2017; Bhattacharyya et al., 2018; Calabrese and Porporato, 2019; Lin et al., 2021), and hyporheic zones (Graham et al., 2017; Stegen et al., 2018; Garayburu-Caruso et al., 2020) or along spatial gradients in soil profiles and within aggregates (Ebrahimi and Or, 2016). There is growing interest in the bioenergetic regulation of OM decomposition under anoxic conditions in soils and aquatic systems because of the potential implications for greenhouse gas emissions and C-storage (Keiluweit et al., 2016; Garayburu-Caruso et al., 2020). For example, the formation of anoxic microsites in rapidly fluctuating redox environments in soils could temporarily inhibit the decomposition of more reduced forms of OM, promoting C storage (Boye et al., 2017; Keiluweit et al., 2017).

The chemical composition of OM determines its energy content, but also poses constraints on C-availability as C released *via* extracellular enzymatic reactions depends on OM chemistry. Reflecting this role, OM chemistry is traditionally used in soil C cycling models to separate C-compartments, but less is known on how the energy content affects microbial processes. Novel molecular methods such as Fourier-transform ion cyclotron resonance (FTICR) mass spectrometry or nuclear magnetic resonance (NMR) characterize the elemental composition of OM to unprecedented levels, posing new challenges on how to incorporate these data into soil C cycling models (Boye et al., 2017; Ding et al., 2020). Recently, Song et al. (2020) presented a substrate explicit decomposition model using data from high-resolution OM characterization that coupled Gibbs energy and C-balance during catabolic and anabolic processes to estimate microbial growth rate. However, such detailed chemical characterization of OM and its effect on metabolism might not be enough to predict changes in microbial growth rates when nutrients are also limited.

Microorganisms are often considered to grow at a fixed chemical composition (homeostatic assumption) so that their ratios of C to other elements do not change when the elemental composition of their substrates varies, at least at the microbial community level (Fanin et al., 2013; Mooshammer et al., 2014b; Schleuss et al., 2019). It is generally assumed that during growth, microorganisms try to meet their nitrogen (N)-demand (i.e., growth rate/microbial C:N) using N from OM (thus releasing excess N *via* ammonification) and compensate for possible N-imbalances using inorganic N (N_Inorg_)-sources (immobilization). When the supply rate of N_Inorg_ is lower than the rate of microbial N-demand, N-limitation ensues (Wutzler et al., 2017). Microorganisms have adapted to deal with N-limited conditions. For example, they could increase the rate of respiration by an overflow respiration mechanism or reduce the uptake of OM by inhibiting extracellular enzyme production (Sistla et al., 2012; Mooshammer et al., 2014a; Manzoni et al., 2021). Depending on the availability of external N_Inorg_ and the C:N ratio of OM, stoichiometric theory quantifies the growth rate as conditions shift between C- and N-limitations (Sterner and Elser, 2002; Sinsabaugh et al., 2013; Manzoni et al., 2017). However, how C- and N-limitations vary depending on the energy content of the OM remains to be studied.

Also, the type of N_Inorg_-source controls microbial growth rate. Microorganisms require N in the form of ammonium (${\text{NH}}_{4}^{+}$) for any cell functions (e.g., protein synthesis); therefore, if the N_Inorg_-source is of a more oxidized form such as nitrate (${\text{NO}}_{3}^{-}$) or nitrite (${\text{NO}}_{2}^{-}$), it must be first reduced to ${\text{NH}}_{4}^{+}$ to be used (Stouthamer, 1977; Lin and Stewart, 1997; Kuypers et al., 2018). This reduction reaction has an energetic cost, because some of the electrons from the catabolism of OM must be allocated to the reduction of the N-source.

Therefore, the availabilities of C, N, and energy lead to different patterns of resource-limitation, which affect microbial growth and respiration. For example, Garayburu-Caruso et al. (2020) showed a shift in the regulation of respiration rate from energy- to N-availability for C- *vs.* N-limited systems under oxic conditions. The respiration rate was thermodynamically regulated because it increased with decreasing degree of reduction of OM as long as N was abundant. In contrast, in N-limited conditions, respiration decreased with the N-content of OM, suggesting that the respiration rate was controlled by N-availability. This result indicates that the energetic constraints on microbial metabolism may become less critical in N-limited systems.

**FIGURE 1:**
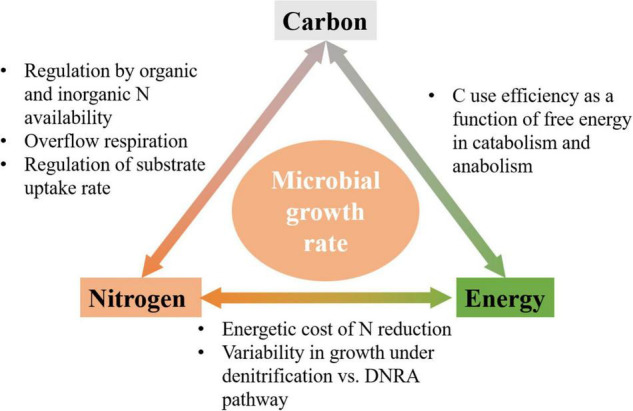
Schematic of links among C, N, and energy flows, and mechanisms by which they affect microbial growth rate.

To further complicate the picture, under anoxic conditions, different N compounds such as ${\text{NO}}_{3}^{-}$, ${\text{NO}}_{2}^{-}$, NO, or N_2_O can act as both EA to drive the catabolism of OM and N-source for microbial growth (Kraft et al., 2014; Kuypers et al., 2018). One such example is the reduction of ${\text{NO}}_{3}^{-}$ *via* denitrification (${\text{NO}}_{3}^{-}$ to N_2_) and the dissimilatory nitrate reduction to ammonia (DNRA) (${\text{NO}}_{3}^{-}$ to ${\text{NH}}_{4}^{+}$). Indeed, the activity of denitrifiers is higher than that of microorganisms performing DNRA at high N_Inorg_ concentrations or in C-limited conditions, where OM already provides N for growth, and *vice versa* at low N_Inorg_ concentrations (Strohm et al., 2007; van den Berg et al., 2016; Putz et al., 2018). These examples illustrate the complex links between C-, N-, and energy-limitations, which we study here from a theoretical perspective (Figure 1).

In this contribution, we build on the existing bioenergetics and stoichiometry theories to develop a theoretical framework for microbial growth under combined C–N–energy limitations. In particular, by integrating stoichiometry and bioenergetic theory of microbial growth, we present a generalized description of microbial growth rate [extending the work by Song et al. (2020)] and address the following questions:

1. Carbon-limitation *vs.* energy-limitation: What are the effects of OM degree of reduction and EA energy yield on microbial growth?
2. Nitrogen-limitation *vs.* energy-limitation: What are the effects of different N_Inorg_ (${\text{NH}}_{4}^{+}$ or ${\text{NO}}_{3}^{-}$)-sources or using N_Inorg_ (${\text{NO}}_{3}^{-}$) as both EA and N-source on microbial growth?
3. Carbon-, Nitrogen-, and energy-limitations: What are the effects of combined thermodynamic (OM degree of reduction and EA energy yield) and biogeochemical factors (OM C:N ratio and N_Inorg_-availability) on microbial growth?

After addressing these questions, we discuss the importance of accounting for limitations in C, N, and energy to explain the variability in growth rate across environmental conditions.

## The C-, N-, and Energy-Limitations of Microbial Growth

### General Assumptions and Macrochemical Equations

Microorganisms are open systems that constantly exchange matter and energy with their surroundings, thereby requiring a non-equilibrium approach to study their dynamics (Prigogine, 1967; Westerhoff et al., 1982; Ornes, 2017). The rates of transformation of mass in a non-equilibrium system depend on the Gibbs energy change, as opposed to systems in equilibrium where the Gibbs energy change mainly describes the feasibility of a process (Bauchop and Elseden, 1960; Jin and Bethke, 2007; LaRowe et al., 2012). Additionally, the coupling of catabolic and anabolic processes is energy dependent, making the microbial CUE a function of the Gibbs energy changes (von Stockar et al., 2008). While we do account for the energy-limitation on CUE, we neglect thermodynamic constraints on microbial-uptake rate (Boudart, 1976; Jin and Bethke, 2007; LaRowe et al., 2012). These constraints are at play only at very low C-availabilities, when only microbial maintenance demand can be met (Hoehler and Jørgensen, 2013; Bradley et al., 2020)—these severely C- and energy-limited systems are not considered here, where we instead focus on conditions that allow for microbial growth.

For simplicity, we take a bioenergetic (macrochemical) perspective on microbial growth and describe growth *via* the coupling of catabolic and anabolic reactions instead of describing individual metabolic pathways. Catabolic and anabolic reactions can be further broken down in several chains of reactions (LaRowe and Amend, 2015, 2016, 2019); however, we simplify the problem and only consider the overall reactants and products, i.e., oxidation of a single OM in catabolism and biosynthesis in anabolism. We assume that an extracellular breakdown of polymeric OM has already occurred, and the microorganisms take up low molecular weight OM available in the surroundings of their cells. Moreover, we assume that homeostasis for microbial growth, i.e., the microorganisms are described as a chemical entity (denoted by B) with fixed elemental ratios [we assumed a microbial biomass C:N ratio *CN*_*B*_ = 5, Roels (1980a)]. If OM contains N, then microbial N-demand is first met by taking up the organic N-source; otherwise, an external N_Inorg_-source must be immobilized (Manzoni et al., 2017; Wutzler et al., 2017). As microbes require N in the form of ${\text{NH}}_{4}^{+}$, more oxidized forms of the N_Inorg_-source must be reduced to ${\text{NH}}_{4}^{+}$ before being converted into biomass (Tiedje et al., 1981).

Microorganisms use OM that acts as both electron donor and C-source with a given C:N ratio (*CN*_*OM*_) and degree of reduction (γ_*OM*_). For a given N_Inorg_-source and EA, we can write a general metabolic equation for microbial growth as follows:


(1) $$\begin{array}{c} \text{OM}+\nu_{EA}\text{EA}+\nu_{N}{\text{N}}_{\text{Inorg}}\to eB+\left(1-e\right){\text{OM}}_{\text{ox}}\\ +\nu_{EA_{red}}E{\text{A}}_{\text{red}}, \end{array}$$

where νis′ are the stoichiometric coefficients of the reactant and product species, N_Inorg_ is the inorganic N source, *e*is the CUE, and OM_ox_ and EA_red_ are the oxidized and reduced forms of electron donors (OM) and acceptors, respectively (all symbols are listed and explained in Table 1). For example, under oxic conditions with O_2_ as the EA, bicarbonate ion and water are OM_ox_ and EA_red_, respectively.

**TABLE 1:** List of symbols and acronyms with their descriptions and units.

Symbol	Description	Unit
γ_*B*_	Degree of reduction of 1 C-mol of OM	e^−^ mol (C-mol OM)^–1^
γ_*OM*_	Degree of reduction of 1 C-mol of biomass	e^−^ mol (C-mol B) ^–1^
γ_*EA*_	number of moles of electrons accepted when reducing 1 mol of EA	e^−^ mol (mol EA) ^–1^
γ_*N*_	number of moles of electrons accepted when reducing 1 N-mol of inorganic N-source	e^−^ mol (N-mol) ^–1^ N-source
Δ_*ana*_*G*_*B*_	Change in Gibbs energy of anabolism for 1 C-mol biomass	kJ (C-mol B)^–1^
Δ_*C*_*G*_*B*_	Change in Gibbs energy of combustion of 1 C-mol biomass	kJ (C-mol B)^–1^
Δ_*C*_*G*_*OM*_	Change in Gibbs energy of combustion of 1 C-mol OM	kJ (C-mol OM) ^–1^
Δ_*cat*_*G*_*OM*_	Change in Gibbs energy of catabolism of 1 C-mol OM	kJ (C-mol OM)^–1^
Δ_*ox*_*G*_*OM*_	Change in Gibbs energy of half-reaction of oxidation of OM	kJ (C-mol OM)^–1^
Δ_*red*_*G*_*EA*_	Change in Gibbs energy of half-reaction of reduction of EA	kJ (mol EA)^–1^
Δ_*red*_*G*_*N*_	Change in Gibbs energy of half-reaction of reduction of inorganic N-source	kJ (N-mol)^–1^ N-source
Δ_*r*_*G*_*B*_	Change in Gibbs energy of overall metabolic reaction for 1 C-mol biomass	kJ (C-mol B)^–1^
Δ_*r*_*G*_*OM*_	Change in Gibbs energy of overall metabolic reaction for 1 C-mol OM-uptake	kJ (C-mol OM)^–1^
ν_*EA*_	Stoichiometry of EA in overall metabolic reaction	mol EA (C-mol OM)^–1^
ν_*EA*_*red*__	Stoichiometry of reduced form of EA in overall metabolic reaction	mol EA_red_ (C-mol OM)^–1^
ν_*N*_	Stoichiometry of inorganic N-source in overall metabolic reaction	N-mol (C-mol OM)^–1^
$\nu_{N}^{ana}$	Stoichiometry of inorganic N-source in anabolism	N-mol (C-mol B)^–1^
B	Biomass (B used as an acronym and chemical species)	-
*CN_B_*	Molar C to N ratio of microbial biomass, *CN*_*B*_ = 5 used in calculation based on chemical formula CH_1.8_O_0.5_N_0.2_ (Roels, 1980a)	C-mol (N-mol)^–1^
*CN*_*OM*_	Molar C to N ratio of the organic matter	C-mol (N-mol)^–1^
*e*	Carbon use efficiency (CUE)	C-mol B (C-mol OM)^–1^
EA	Electron acceptor (EA used as an acronym and chemical species)	-
EA_red_	Reduced form of electron acceptor	-
*G_C_*	C-limited growth rate	C-mol B day^–1^
*G_N_*	N-limited growth rate	C-mol B day^–1^
*G*_*norm*_	Normalized growth rate, $G_{norm}=\frac{G}{U_{OM}}$	-
*I_N_*	Rate of inorganic nitrogen supply	N-mol day^–1^
*I*_*norm*_	Normalized *I_N_*, $I_{norm}=\frac{I_{N}}{U_{OM}}$	N-mol (C-mol OM)^–1^
N_Inorg_	Inorganic nitrogen (N_Inorg_used as an acronym and chemical species)	
DNRA	Dissimilatory nitrate reduction to ammonia	Acronym
OM	Organic matter (OM used as an acronym and chemical species)	Acronym
OM_ox_	Oxidized form of organic matter	-
*n_i_*	Numbers of elements in OM compounds (*i* = *C*, *H*, *N*, *O*); *i* = *z* is the overall charge	-
TER	Threshold elemental ratio	C-mol (N-mol)^–1^
*U*_*OM*_	Uptake rate of the organic matter	C-mol OM day^–1^
*x*_*EA*_	Stoichiometry of EA in catabolism	mol EA (C-mol OM)^–1^
*x_N_*	Stoichiometry of inorganic N in catabolism	N-mol N-source (C-mol OM)^–1^
*y_N_*	Stoichiometry of inorganic N when nitrate is the EA and N-source	N-mol (C-mol OM)^–1^

**FIGURE 2:**
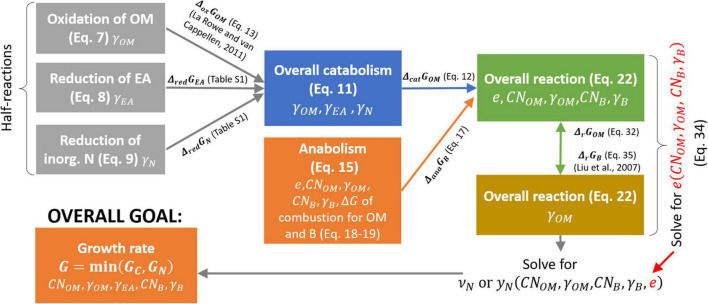
Roadmap for the calculation of normalized microbial growth rate (*G*) as a function of OM and microbial C:N ratios (*CN*_*OM*_ and *CN_B_*, respectively), and degrees of reduction of OM, EA, and biomass (γ_*OM*_,γ_*EA*_,and γ_*B*_, respectively). Each box refers to a half or overall reaction (with equation number in brackets) and includes the parameters affecting the reaction stoichiometry (listed at the bottom of each box). Arrows represent the changes in Gibbs energy associated to each half reaction, which is then used to calculate the changes in Gibbs energy of the overall reactions. Symbols are defined in Table 1.

When EA and the N_Inorg_-source are the same such as in denitrification or DNRA pathways, the metabolic equation for microbial growth can be written as follows:


(2) $$\text{OM}+y_{N}{\text{N}}_{\text{Inorg}}\to eB+\left(1-e\right){\text{OM}}_{\text{ox}}+\nu_{EA_{red}}E{\text{A}}_{\text{red}},$$

where *y_N_* is the stoichiometric coefficient of the N_Inorg_-source, which is different in Eqs. (1) and (2). In denitrification or DNRA pathways, the N-source is ${\text{NO}}_{3}^{-},$which is reduced to EA_red_; i.e., either N_2_ or ${\text{NH}}_{4}^{+}$, respectively. Note that to balance the chemical reactions throughout the text, H^+^ and H_2_O with appropriate stoichiometry must be added on either side; however, including H^+^ and H_2_O is not necessary for our purposes, so we only balance C and N in the reactions. After defining the reaction rate (section “Microbial Growth Rate Under C- and N-Limitations”), in section “Bioenergetics of Microbial Growth” we formulate the stoichiometric coefficients of Eqs. (1) and (2) as the functions of microbial and OM C:N ratios, and the degrees of reduction of biomass, OM, and EA. In this way, these coefficients are calculated under a range C-, N-, and energy-limited conditions. A schematic of the bioenergetic framework is provided in Figure 2.

### Microbial Growth Rate Under C- and N-Limitations

Using the metabolic Eqs. (1) and (2), we now formulate the microbial growth rate under C- and N-limited conditions by integrating stoichiometry theory and bioenergetics. As Eqs. (1) and (2) are written with respect to the uptake of 1 C-mol of OM, the rate of reaction is the same as the uptake rate of OM, denoted in C-limited conditions as *U*_*OM*_ with units of C-mol OM day^–1^. The microbial growth rate *G_C_* under C-limitation can be written as follows:


(3) $$G_{C}=eU_{OM},$$

where the subscript C in *G*_*C*_ denotes microbial growth rate under C-limitation, and *e*is the maximum CUE without considering maintenance costs (as mentioned above, we focus on conditions where growth can occur and maintenance respiration is relatively small). When the N_Inorg_-source is a limiting reactant, then the metabolic reaction rate is controlled by the N supply, and growth becomes N-limited. By dividing Eqs. (1) and (2) with the stoichiometric coefficient of the N_Inorg_-source (ν_*N*_ or *y_N_*) and taking the rate of N_Inorg_ supply as the rate of the metabolic reaction, we can write the microbial growth rate under N-limited condition as follows:


(4) $$G_{N}=\left\{ \begin{array}{c} \frac{e}{\nu_{N}}I_{N},\mathrm{when}\mathrm{EA}\mathrm{and}\text{N-source}\mathrm{are}\mathrm{different};\mathrm{Eq}.\left(1\right)\\ \frac{e}{y_{N}}I_{N},\text{when}\mathrm{EA}\mathrm{and}\text{N-source}\mathrm{are}\mathrm{the}\mathrm{same};\mathrm{Eq}.\left(2\right) \end{array}\right.,$$

where the subscript N in *G*_*N*_ denotes growth rate under N-limitation, and *I_N_* is the rate of supply of the N_Inorg_-source. Note that the units of *G_C_* and *G_N_* are the same, C-mol B day^–1^.

A general form of microbial growth rate (*G*) can then be written by taking the minimum of *G_C_* and *G_N_* as defined by Eq. (5) (Liebig’s law),


(5) $$G=\min\left(G_{C},G_{N}\right).$$

Here, we do not focus on the functional form of uptake kinetics, rather on using bioenergetics to model growth rate and stoichiometric constraints for given *U*_*OM*_. Hence, we normalize by *U*_*OM*_ both growth rate $\left(G_{norm}=\frac{G}{U_{OM}}\right)$ and N_Inorg_ uptake rate $\left(I_{norm}=\frac{I_{N}}{U_{OM}}\right)$. As a result, the non-dimensional growth rate becomes


(6) $$G_{norm}=\left\{ \begin{array}{c} e\min\left(1,\frac{I_{norm}}{\nu_{N}}\right),\\ \mathrm{when}\mathrm{EA}\mathrm{and}\text{N-source}\mathrm{are}\mathrm{different};\mathrm{Eq}.\left(1\right)\\ e\min\left(1,\frac{I_{norm}}{y_{N}}\right),\\ \mathrm{when}\mathrm{EA}\mathrm{and}\text{N-source}\mathrm{are}\mathrm{the}\mathrm{same};\mathrm{Eq}.\left(2\right) \end{array}\right.$$

In the following sections, we consider the coupling of catabolic and anabolic reactions based on their Gibbs energy balances, and account for the energy cost to reduce N_Inorg_ to estimate both CUE and the stoichiometric coefficient for N_Inorg_ (“overall goal” in Figure 2).

### Bioenergetics of Microbial Growth

The two general reactions in Eqs. (1) and (2) are here broken down into catabolic and anabolic reactions, and these are further broken down into oxidation and reduction half-reactions (Figure 2). This detailed formulation allows calculating the stoichiometric coefficients in Eqs. (1) and (2) as a function of OM and N-source characteristics.

#### Catabolism of Organic Matter

Without the loss of generality, the macrochemical catabolic reaction is formulated for one C-mol of OM. When OM does not contain any N or have high C:N ratios (N-limited), microorganisms immobilize N_Inorg_ from the environment. If this N_Inorg_-source is not ${\text{NH}}_{4}^{+}$, then some of the electrons produced from the oxidation of the OM [Eq. (7)] must be allocated to the reduction of the N_Inorg_-source. This allocation is often performed by intracellular electron carrier proteins, either NADP(H) or ferredoxins (Bloom, 2010). Therefore, the energetic cost of the reduction of the N_Inorg_-source to ammonium must be accounted for in the catabolic reaction. The catabolic reaction is formulated by considering the half-reaction of oxidation of OM and reduction of EA and N_Inorg_. To keep the formulation general, we separately consider the redox reactions for OM and EAs (top left in Figure 2).

The half-reaction of oxidation of the OM can be written as follows:


(7) $$\text{OM}\to {\text{OM}}_{\text{ox}}+\frac{1}{CN_{OM}}{\text{NH}}_{4}^{+}+\gamma_{OM}{\text{e}}^{-}+\Delta_{ox}G_{OM},$$

where OM_ox_ is the oxidized form of OM, 1/*CN*_*OM*_ is the stoichiometric coefficient for ${\text{NH}}_{4}^{+}$ released during the oxidation, γ_*OM*_ is the degree of reduction of the OM, and Δ_*ox*_*G*_*OM*_ [kJ (C-mol OM)^–1^] is the change in Gibbs energy of the oxidation reaction. Note that for all Gibbs energy changes, the subscript of Δ indicates the reaction and the subscript of *G* is the substance per unit of which the Gibbs energy is reported. For simplicity, we assume that OM_ox_ is always bicarbonate and catabolism does not produce any other organic product.

Next, the reduction half-reaction of a generic EA can be written as follows:


(8) $$\text{EA}+\gamma_{EA}{\text{e}}^{-}\to \nu_{EA_{red}}E{\text{A}}_{\text{red}}+\Delta_{red}G_{EA},$$

where γ_*EA*_ is the number of moles of electron received by the EA, EA_red_ is the reduced form of EA (e.g., H_2_O in case of O_2_), and ν_*EA*_*red*__ is its stoichiometric coefficient; Δ_*red*_*G*_*EA*_ [kJ (mol EA)^–1^] is the change in Gibbs energy of the reduction reaction. A list of commonly occurring half-reactions of reduction of EAs is provided in Supplementary Table 1.

Next, the reduction of the N_Inorg_-source to ammonium is given by


(9) $${\text{N}}_{\text{Inorg}}+\gamma_{\text{N}}e^{-}\to {\text{NH}}_{4}^{+}+\Delta_{red}G_{N},$$

where γ_*N*_ and Δ_*red*_*G*_*N*_ are, respectively, the number of moles of electrons accepted when reducing 1 N-mol of N_Inorg_-source and the change in Gibbs energy of the reaction to reduce the N-source. For ${\text{NH}}_{4}^{+}$ as N_Inorg_-source, no reduction would be required and γ_*N*_ = 0. The amount of N-source reduced to ${\text{NH}}_{4}^{+}$ depends on the microbial N-demand for growth, which in turn depends on the CUE and the C:N ratio of the OM. If *x_N_* is the number of moles of N_Inorg_ reduced to ${\text{NH}}_{4}^{+}$, *x_N_* × γ_*N*_ moles of electron per mol of OM must be transferred from the oxidation of OM to the reduction of the N-source in Eq. (9).

Finally, the catabolic reaction is obtained by adding the three half-reactions, Eqs. (7)–(9), and adjusting the stoichiometric coefficients so that the electrons are balanced (top center of Figure 2),


(10) $$Eq.\left(7\right)+x_{N}\times Eq.\left(9\right)+\frac{\gamma_{OM}-x_{N}\gamma_{N}}{\gamma_{EA}}\times Eq.\left(8\right)=0,$$

(11) $$\begin{array}{c} \text{OM}+x_{EA}\text{EA}+x_{N}{\text{N}}_{\text{Inorg}}\to {\text{OM}}_{\text{ox}}+\nu_{EA_{red}}x_{EA}{\text{EA}}_{\text{red}}\\ +\left(x_{N}+\frac{1}{CN_{OM}}\right){\text{NH}}_{4}^{+}+\Delta_{cat}G_{OM}, \end{array}$$

where $x_{EA}=\frac{\gamma_{OM}-x_{N}\gamma_{N}}{\gamma_{EA}}$ is the stoichiometric coefficient of the EA; Δ_*cat*_*G*_*OM*_ [kJ (C-mol OM)^–1^] is the amount of Gibbs energy released by catabolizing 1 C-mol of OM for a given EA. Similar to Eq. (11), Δ_*cat*_*G*_*OM*_ is given by summing the Gibbs energy of the three half-reactions, Eqs. (7)–(9), multiplied by appropriate stoichiometric coefficients.


(12) $$\Delta_{cat}G_{OM}=\Delta_{ox}G_{OM}+x_{N}\Delta_{red}G_{N}+x_{EA}\Delta_{red}G_{EA}.$$

In principle, Δ_*cat*_*G*_*OM*_ can be calculated directly from Eq. (11) using Hess’s law, if the involved species and their chemical formulae are known. However, such information is often not known, as in the case of plant residues or soil OM. Using Eq. (12) is advantageous because the change in Gibbs energy of oxidation of OM can be estimated based on its degree of reduction γ_*OM*_, as proposed by LaRowe and Van Cappellen (2011),


(13) ΔoxGOM=60.3-28.5(4-γOM)[kJ(C-molOM)]-1.

The empirical formulation of Δ_*ox*_*G*_*OM*_ in Eq. (13) holds under standard conditions, therefore it needs to be modified under non-standard OM aqueous concentrations [for more details, see LaRowe et al. (2012) and Song et al. (2020)]. The γ_*OM*_ of 1 C-mol of OM is estimated based on the OM chemical formula as follows:


(14) $$\gamma_{OM}=\frac{4n_{C}+n_{H}-3n_{N}-2n_{O}-n_{z}}{n_{C}},$$

where *n_i_* for *i* = *C*, *H*, *N*, or *O* are the numbers of th*ei*_th_ element and *z* is the overall charge in the chemical formula of OM. While the actual values of γ_*OM*_ can be calculated from the chemical formula of OM, here we explore a range of values from 0 (least reduced, CO_2_) to 8 (most reduced, CH_4_), to cover the whole spectrum of organic compounds.

The change in Gibbs energy of the reduction reactions (Δ_*red*_*G*_*EA*_ and Δ_*red*_*G*_*N*_, Supplementary Table 1) are calculated using standard Gibbs energy of formation listed in Supplementary Table 3. With this information, Δ_*cat*_*G*_*OM*_ can be estimated from the degree of reduction of the OM. An example of the calculations for the catabolism of glycine with ${\text{NO}}_{3}^{-}$ as both EA and the N-source, and of glucose with O_2_ as the EA and ${\text{NO}}_{3}^{-}$ as the N-source, are provided in Supplementary Table 2.

#### Anabolism of Microbial Biomass

Energy from catabolism is then transferred to the anabolism to support the synthesis of new biomass (center of Figure 2). Here, we formulate a generic reaction for the anabolism (biosynthesis) for 1 C-mol of microbial biomass. There are multiple ways of writing a macrochemical representation of an anabolic reaction (von Stockar et al., 2008; Battley, 2009; Kleerebezem and Van Loosdrecht, 2010). We follow Battley (2009), who simplifies Gibbs energy calculations by neglecting EA in anabolism. Briefly, 1 C-mol of biomass is formed from the same organic C used in catabolism and ${\text{NH}}_{4}^{+}$ as the N-source [other N-sources are reduced to ${\text{NH}}_{4}^{+}$ before being used, Eq. (9)]. Further, a degree of reduction balance is used to balance the electrons, and the imbalance of C is balanced by adding OM_ox_(i.e., bicarbonate) to either side of the reaction. Thus, the anabolic reaction for an OM with given *CN*_*OM*_ can be written as follows:


(15) $$\frac{\gamma_{B}}{\gamma_{OM}}\text{OM}+\nu_{N}^{ana}{\text{NH}}_{4}^{+}\to \text{B}+\left(\frac{\gamma_{B}}{\gamma_{OM}}-1\right){\text{OM}}_{\text{ox}}+\Delta_{ana}G_{B},$$

where γ_*B*_ = 4.2 is the degree of reduction of the microbial biomass and Δ_*ana*_*G*_*B*_ is the change in Gibbs energy of anabolism. Balancing N on both sides yields the stoichiometric coefficient of the N-source (i.e., ${\text{NH}}_{4}^{+}$),


(16) $$\nu_{N}^{ana}=\frac{1}{CN_{B}}-\frac{\gamma_{B}}{\gamma_{OM}}\frac{1}{CN_{OM}}.$$

The value of Δ_*ana*_*G*_*B*_ [kJ (C-mol B)^–1^] is estimated by writing the Gibbs energy balance of Eq. (15), as follows:


(17) $$\Delta_{ana}G_{B}=\frac{\gamma_{B}}{\gamma_{OM}}\Delta_{C}G_{OM}-\Delta_{C}G_{B},$$

where Δ_*C*_*G*_*OM*_ and Δ_*C*_*G*_*B*_ are the changes in Gibbs energy of combustion for a given EA. Note that we have used combustion as the reference state so that only the organic C appears in Eq. (17).

To find Δ_*C*_*G*_*OM*_ and Δ_*C*_*G*_*B*_, we can regard a catabolic reaction as analogous to a combustion reaction when a N_Inorg_-source is not included. Therefore, for *x*_*N*_ = 0 and O_2_ as the EA, Eq. (11) represents the complete combustion of an organic C compound to CO_2_. Thus, from Eqs. (12) and (13), we obtain Δ_*C*_*G*_*OM*_ and Δ_*C*_*G*_*B*_ as follows:


(18) $$\Delta_{C}G_{OM}=\left(28.5\gamma_{OM}-53.7\right)+\frac{\gamma_{OM}}{\gamma_{EA}}\Delta_{red}G_{EA},$$

(19) $$\Delta_{C}G_{B}=\left(28.5\gamma_{B}-53.7\right)+\frac{\gamma_{B}}{\gamma_{EA}}\Delta_{red}G_{EA}.$$

Now, from Eqs. (17)–(19), we obtain Δ_*ana*_*G*_*B*_ as follows:


(20) $$\Delta_{ana}G_{B}=53.7\left(1-\frac{\gamma_{B}}{\gamma_{OM}}\right).$$

Examples of the anabolic reaction on glycine and glucose are provided in Supplementary Table 2.

#### Overall Metabolic Reaction

The overall metabolic reaction is obtained by summing the catabolic and anabolic reactions so that 1 C-mol of OM is used to form *e* C-mol of biomass (right of Figure 2). Thus, multiplying the catabolic reaction Eq. (11) by $1-e\frac{\gamma_{B}}{\gamma_{OM}}$ and the anabolic reaction Eq. (15) by *e*, and summing these reactions, gives the overall metabolic reaction of microbial growth as follows:


(21) $$\left(1-e\frac{\gamma_{B}}{\gamma_{OM}}\right)\times \mathrm{catabolism}+e\times \mathrm{anabolism}=0,$$

(22) $$\begin{array}{c} \text{OM}+\nu_{EA}\text{EA}+\nu_{NH_{4}}{\text{NH}}_{4}^{+}+\nu_{N}{\text{N}}_{\text{Inorg}}\to eB\\ +\left(1-e\right){\text{OM}}_{\text{ox}}+\nu_{EA_{red}}{\text{EA}}_{\text{red}}+\Delta_{r}G_{OM}. \end{array}$$

In Eq. (22), Δ_*r*_*G*_*OM*_ is the Gibbs energy change for the overall metabolic reaction, and ν_*i*_ are the stoichiometric coefficients of each reaction species (*i* = EA, EA_*red*_, ${\text{NH}}_{4}^{+}$, N_Inorg_). Equation (22) thus recovers the form of Eqs. (1) or (2), but now all stoichiometric coefficients can be determined as a function of the degrees of reduction of OM, EA, and biomass, and the C:N ratios of OM and biomass,


(23) $$\nu_{EA}=\left(1-e\frac{\gamma_{B}}{\gamma_{OM}}\right)x_{EA}=\left(1-e\frac{\gamma_{B}}{\gamma_{OM}}\right)\frac{\gamma_{OM}-x_{N}\gamma_{N}}{\gamma_{EA}},$$

(24) $$\nu_{NH_{4}}=e\nu_{N}^{ana}-\left(1-e\frac{\gamma_{B}}{\gamma_{OM}}\right)\left(x_{N}+\frac{1}{CN_{OM}}\right),$$

(25) $$\nu_{N}=\left(1-e\frac{\gamma_{B}}{\gamma_{OM}}\right)x_{N},$$

(26) $$\nu_{EA_{red}}=\left(1-e\frac{\gamma_{B}}{\gamma_{OM}}\right)x_{EA}.$$

In Eqs. (23)–(26), *x_N_* is not known, but it can be calculated by setting ν_*NH*_4__ = 0, because ${\text{NH}}_{4}^{+}$ used for microbial growth is already accounted for in the stoichiometric coefficient of the N-source. Thus, setting ν_*NH*_4__ = 0 yields the following:


(27) $$x_{N}=\left(\frac{e}{CN_{B}}-\frac{1}{CN_{OM}}\right){\left(1-e\frac{\gamma_{B}}{\gamma_{OM}}\right)}^{-1}.$$

The expressions for ν_*EA*_ and ν_*N*_ can now be simplified by inserting *x_N_* in Eqs. (23) and (25), which are expressed as follows:


(28) $$\nu_{EA}=\frac{1}{\gamma_{EA}}\left[\gamma_{OM}-e\gamma_{B}-\gamma_{N}\left(\frac{e}{CN_{B}}-\frac{1}{CN_{OM}}\right)\right],$$

(29) $$\nu_{N}=\frac{e}{CN_{B}}-\frac{1}{CN_{OM}}.$$

Equations (28) and (29) can be used to assess N-demand for microbial growth under oxic or anoxic conditions for any EA. Under C-limited, oxic conditions and assuming ${\text{NH}}_{4}^{+}$ is the N-source, the rate of N-uptake, ν_*N*_*U*_*OM*_, is the same as given by Manzoni et al. (2017). By setting Eq. (29) equal to zero, we can calculate the threshold C:N ratio of the OM at which no ${\text{NH}}_{4}^{+}$ is formed or immobilized. Above this C:N ratio—often called threshold elemental ratio (TER)—microbial growth becomes limited by the supply of organic N, which is expressed as follows:


(30) $$TER=\frac{CN_{B}}{e}.$$

If *CN*_*OM*_ < *TER*, then ν_*N*_ > 0 and net release of ${\text{NH}}_{4}^{+}$ occurs (i.e., net N mineralization), whereas if *CN*_*OM*_ > *TER*, then ν_*N*_ < 0 and net uptake of ${\text{NH}}_{4}^{+}$ occurs (i.e., net immobilization). Moreover, if the supply of N_Inorg_, *I_N_*, cannot sustain the required rate of immobilization, the microbial growth rate is determined by the rate of supply of the external N_Inorg_-source; this condition is denoted as N-limitation (net required immobilization rate > *I_N_*). Under N-limited conditions, microorganisms reduce the C-uptake rate, thereby decreasing their growth rate so that N-demand matches N_Inorg_-availability (i.e., *I_N_*). Mathematically, the reduction in *U*_*OM*_ is obtained from Eq. (6) as a function of the stoichiometry of N in the overall metabolic reaction, which in turn is a function of *CN*_*OM*_ and *I*_*norm*_.

If EA and N_Inorg_-source are the same (i.e., ${\text{NO}}_{3}^{-}$), such as during denitrification or DNRA, N is used for both oxidation of OM and microbial N-demand for growth; therefore, N-limitation would affect both uptake of OM and microbial growth. Thus, the total amount of N used [*y*_*N*_ in Eq. (2)] is given as the sum of ν_*EA*_ and ν_*N*_, as follows:


(31) $$y_{N}=\nu_{EA}+\nu_{N}.$$

Examples of the overall metabolic reaction on glycine and glucose are provided in Supplementary Table 2.

#### Gibbs Energy Change of Metabolic Reaction and Carbon Use Efficiency

The change in Gibbs energy of the metabolic reaction, Δ_*r*_*G*_*OM*_ [kJ (C-mol)^–1^ OM], can be obtained by adding catabolic and anabolic reactions as done in Eq. (21), and can be written as follows:


(32) $$\Delta_{r}G_{OM}=\left(1-e\frac{\gamma_{B}}{\gamma_{OM}}\right)\Delta_{cat}G_{OM}+e\Delta_{ana}G_{B}.$$

Equation (32) can be used to estimate Δ_*r*_*G*_*OM*_ knowing the CUE, or inversely it can be used to estimate CUE if Δ_*r*_*G*_*OM*_ is known. It has been shown that the energy dissipated from microbial systems can be predicted by the degree of reduction of the OM, γ_*OM*_ (Heijnen and Dijken, 1992; Liu et al., 2007). However, these formulations are based on the Gibbs energy dissipated to produce 1 C-mol of biomass (Δ_*r*_*G*_*B*_); therefore, we divide Eq. (32) by CUE and obtain the following:


(33) $$\Delta_{r}G_{B}=\left(\frac{1}{e}-\frac{\gamma_{B}}{\gamma_{OM}}\right)\Delta_{cat}G_{OM}+\Delta_{ana}G_{B},$$

where $\Delta_{r}G_{B}=\frac{\Delta_{r}G_{OM}}{e}$, Δ_*cat*_*G*_*OM*_ is given by Eq. (12), and Δ_*ana*_*G*_*B*_ by Eq. (20). Simplifying the above equation to obtain CUE as a function of Gibbs energies yields the following:


(34) $$e=\frac{\Delta_{cat}G_{OM}}{\Delta_{r}G_{B}-\Delta_{ana}G_{B}+\frac{\gamma_{B}}{\gamma_{OM}}\Delta_{cat}G_{OM}}.$$

Finally, the only remaining unknown in Eq. (34) is Δ_*r*_*G*_*B*_, which is given by Liu et al. (2007) as follows:


(35) $$-\Delta_{r}G_{B}=\left\{ \begin{array}{c} \frac{666.2}{\gamma_{OM}}+243.1\mathrm{for}\gamma_{OM}\leq 4.67\\ 157\gamma_{OM}-339\mathrm{for}\gamma_{OM}>4.67 \end{array}\right..$$

It is clear from Eqs. (12), (20), and (35) that the microbial CUE depends mainly on the degree of reduction of the OM and microbial biomass, and the type of EAs. Note that in Eq. (34), Δ_*cat*_*G*_*OM*_ is also a function of *e*, making Eq. (34) an implicit non-linear equation in *e*, which therefore needs to be solved numerically. An example of metabolic reaction on glycine and glucose is presented in Supplementary Table 2.

We assumed standard conditions for calculating Gibbs energy, so that the stoichiometry of the metabolic reactions is also representative of standard conditions, i.e., species concentrations are at 1 mol L^–1^, pH 7, temperature of 298 K, and pressure of 1 bar. In a dynamic system, changing concentrations of the involved species, pH, or temperature would change the Gibbs energy of metabolic reactions, and thus also the stoichiometric coefficients of such reactions. Our framework can be generalized by including the effects of non-standard conditions as shown in previous work (Kleerebezem and Van Loosdrecht, 2010; LaRowe and Amend, 2015, 2016; Delattre et al., 2019).

To summarize the theory section, we started with a general description of microbial growth rate under C- or N-limited conditions (section “Microbial Growth Rate Under C- and N-Limitations”). The stoichiometric coefficients needed to calculate growth rate are estimated by splitting the overall metabolic reaction into catabolic and anabolic parts and considering their Gibbs energies (sections “Catabolism of Organic Matter” and “Anabolism of Microbial Biomass”). Finally, the stoichiometric coefficients of the metabolic reaction—mainly CUE (*e*)—and of N-uptake (*v_N_* or *y_N_*) were constrained using bioenergetics (sections “Overall Metabolic Reaction” and “Gibbs Energy Change of Metabolic Reaction and Carbon Use Efficiency”). Table 2 summarizes some simplifications of ν_*EA*_, *v*_*N*_, and *y_N_* under specific conditions for the OM; e.g., when OM contains N or when EA and N_Inorg_-source are both ${\text{NO}}_{3}^{-}$.

**TABLE 2:** Values of the stoichiometric coefficients for the N-source (ν_*N*_) and microbial growth rate (*G*_*N*_), for various types of OM and N_Inorg_-sources.

OM	ν_N_	${\text{G}}_{N}=\frac{e}{\nu_{N}}{\text{I}}_{N}$
OM does not contain N, N-source: ${\text{NH}}_{4}^{+}$$\left(\frac{1}{CN_{OM}}=0,\gamma_{N}=0\right)$	$\frac{e}{CN_{B}}$	*I_N_CN_B_*
OM does not contain N, N-source is not ${\text{NH}}_{4}^{+}$$\left(\frac{1}{CN_{OM}}=0,\gamma_{N}\neq 0\right)$	$\frac{e}{CN_{B}}$	*I_N_CN_B_*
OM contains N, N-source: ${\text{NH}}_{4}^{+}$$\left(\frac{1}{CN_{OM}}\neq 0,\gamma_{N}=0\right)$	$\frac{e}{CN_{B}}-\frac{1}{CN_{OM}}$	$\frac{e}{\frac{e}{CN_{B}}-\frac{1}{CN_{OM}}}I_{N}$
OM contains N, N-source is not ${\text{NH}}_{4}^{+}$$\left(\frac{1}{CN_{OM}}\neq 0,\gamma_{N}\neq 0\right)$	$\frac{e}{CN_{B}}-\frac{1}{CN_{OM}}$	$\frac{e}{\frac{e}{CN_{B}}-\frac{1}{CN_{OM}}}I_{N}$
**EA and N-source:** ${\text{NO}}_{3}^{-}$	**y_N_** = **ν_EA_** + **ν_N_**	${\text{G}}_{N}=\frac{e}{y_{N}}{\text{I}}_{N}$
DNRA pathway: ${\text{NO}}_{3}^{-}$ reduced to ${\text{NH}}_{4}^{+}\left(\gamma_{N}=8,\gamma_{EA}=8\right)$	$\left(1-e\frac{\gamma_{B}}{\gamma_{OM}}\right)\frac{\gamma_{OM}}{\gamma_{N}}$	$\frac{e}{\left(1-e\frac{\gamma_{B}}{\gamma_{OM}}\right)\frac{\gamma_{OM}}{\gamma_{N}}}I_{N}$
Denitrification pathway: ${\text{NO}}_{3}^{-}$ reduced to N_2_(γ_*N*_ = 8, γ_*EA*_ = 5)	$\left(1-e\frac{\gamma_{B}}{\gamma_{OM}}\right)\frac{\gamma_{OM}}{\gamma_{EA}}+\left(1-\frac{\gamma_{N}}{\gamma_{EA}}\right)\left(\frac{e}{CN_{B}}-\frac{1}{CN_{OM}}\right)$	$\frac{e}{\left(1-e\frac{\gamma_{B}}{\gamma_{OM}}\right)\frac{\gamma_{OM}}{\gamma_{EA}}+\left(1-\frac{\gamma_{N}}{\gamma_{EA}}\right)\left(\frac{e}{CN_{B}}-\frac{1}{CN_{OM}}\right)}I_{N}$

## Results

First, we studied the interactions between C- and energy-limitations, showing how the degree of reduction of the OM and the energy-availability from the reduction of the EA (Δ_*red*_*G*_*EA*_) affect the growth rate (section “Interactions Between C- and Energy-Limitations”). Second, we investigated the interactions among C-, N-, and energy-limitations (section “Interactions Among C-, N-, and Energy-Limitations”), when the OM either does not contain N (section “Microbial Growth on Organic Matter Without N”) or it does (section “Microbial Growth on Organic Matter Containing N”). In section “Microbial Growth on Organic Matter Without N,” we showed how the degree of reduction of the OM under oxic conditions and different N_Inorg_-sources (${\text{NO}}_{3}^{-}$ and ${\text{NH}}_{4}^{+}$) and N availabilities affect the microbial growth rate. Next, we focused on growth rate under anoxic conditions when ${\text{NO}}_{3}^{-}$ is both EA and N-source; in this case, ${\text{NO}}_{3}^{-}$ is reduced to ${\text{NH}}_{4}^{+}$ *via* either DNRA or denitrification pathway. In section “Microbial Growth on Organic Matter Containing N,” we studied the microbial growth rate along a gradient of organic N from OM and N_Inorg_-availability. In this case, we used ${\text{NH}}_{4}^{+}$ as N-source, but calculated the growth rate under a range of EAs such as O_2_, Fe^3+^ (goethite), Fe^3+^ (ferrihydrite), and ${\text{SO}}_{4}^{2-}$ (sulfate). For simplicity, in section “Interactions Among C-, N-, and Energy-Limitations,” we have further assumed that the availability of EA does not limit microbial growth.

### Interactions Between C- and Energy-Limitations

**FIGURE 3:**
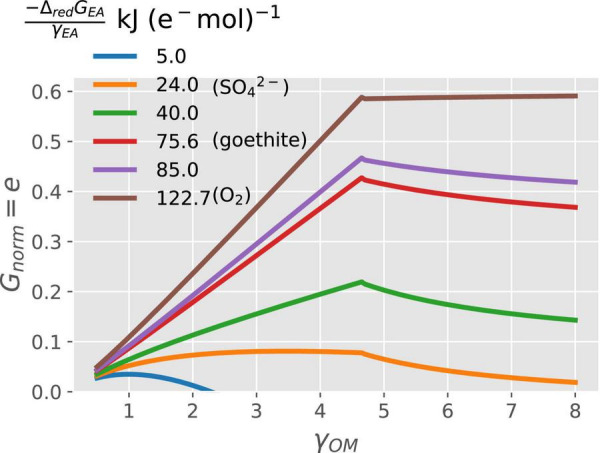
Variation of normalized growth rate (*G*_*norm*_) with the degree of reduction of the OM (γ_*OM*_ on the *x*-axis) and energy available from the reduction of EA (Δ_*red*_*G*_*EA*_; curves with different colors).

In general, when N is not limiting, the normalized growth rate (*G*_*norm*_ = *e*) increases with increasing degree of reduction of OM (γ_*OM*_), except at the high values of γ_*OM*_, when it can also decrease (Figure 3). For a given γ_*OM*_, *G*_*norm*_ also increases proportionally to the change in Gibbs energy of the EA reduction Δ_*red*_[*G*_*EA*_ in kJ (e^−^ mol)^–1^] (different line colors in Figure 3). Within these general trends, specific interactions between γ_*OM*_ and Δ_*red*_*G*_*EA*_ emerge. Under energy rich conditions; i.e., high values of both γ_*OM*_ and |−Δ_*red*_*G*_*EA*_| (e.g., brown curve), the normalized growth rate is maximum because of high CUE. Under energy-limited conditions, the growth increases with increasing γ_*OM*_ as long as γ_*OM*_ < 4.7, but for γ_*OM*_ > 4.7, the growth rate starts decreasing when catabolism is coupled with low energy yielding EA (low values of |−Δ_*red*_*G*_*EA*_|; e.g., orange curve). These combined trends cause the growth rate to attain a peak at an intermediate γ_*OM*_ (blue–red curves). To summarize, purely C-limited conditions only occur at high γ_*OM*_ and |−Δ_*red*_*G*_*EA*_|, while the energetic constraints are at play in all other cases.

### Interactions Among C-, N-, and Energy-Limitations

#### Microbial Growth on Organic Matter Without N

**FIGURE 4:**
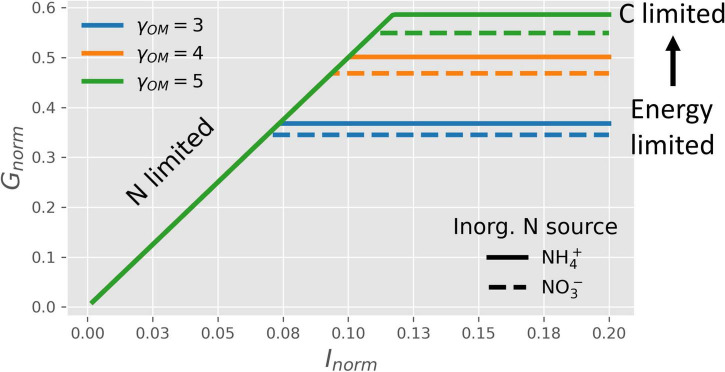
OM without N: normalized microbial growth rate (*G*_*norm*_) under oxic conditions as a function of N_Inorg_-availability (*I*_*norm*_), degrees of reduction of the OM (γ_*OM*_; lines with different colors), and source of N_Inorg_ (solid lines for ${\text{NH}}_{4}^{+}$ *vs.* dashed lines for ${\text{NO}}_{3}^{-}$). Horizontal lines, blue to green, represent the transition from energy to C-limited conditions.

Figure 4 shows how the normalized microbial growth rate varies under C-limitation *vs.* N-limitation under oxic conditions. At high N-availability (high values of *I*_*norm*_), microbes are C-limited and the normalized growth rate only depends on the CUE. In turn, the CUE increases with a higher degree of reduction of the OM, causing the growth rate to increase with γ_*OM*_ (lines with different colors), as also shown in Figure 3. In contrast, at low N-availability (low values of *I*_*norm*_), microbes are N-limited and their growth is constrained by *I*_*norm*_instead of CUE. As a consequence, N-limited growth is independent of the degree of reduction of the OM (Table 2). When microbes are supplied with an N_Inorg_-source other than ${\text{NH}}_{4}^{+}$, the energetic cost for ${\text{NO}}_{3}^{-}$ reduction to ${\text{NH}}_{4}^{+}$ reduces the CUE [dashed vs. solid lines in Figure 4]. Even if Figure 4 shows the normalized growth rate under oxic conditions, the same dependence of the growth rate on N_Inorg_-availability and γ_*OM*_ occurs for any EA also under anoxic conditions, although the lines shift depending on the metabolic pathway of N, as shown next.

**FIGURE 5:**
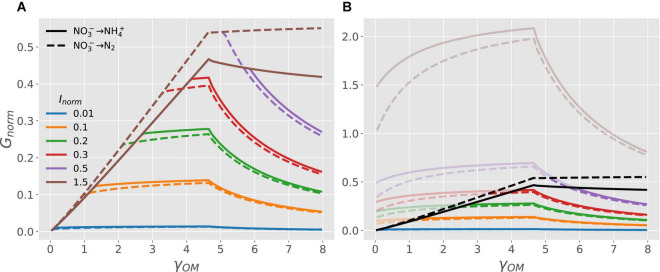
OM without N and nitrate as EA and N-source: **(A)** normalized microbial growth rate (*G*_*norm*_) as a function of N_Inorg_-availability (*I*_*norm*_) and degree of reduction of the OM (γ_*OM*_; curves with different colors), when the OM is catabolized *via* denitrification (dashed curves) or DNRA pathway (solid curves). **(B)** Normalized microbial growth rate under C-limitation [*G*_*C*, *norm*_ = *e*, Eq. (3); black curves] and N-limitation [*G*_*N*, *norm*_ = (*e*/*y*_*N*_)*I*_*norm*_, Eq. (4); colored curves]. The parts of the curves that are not realized [recall that *G*_*norm*_ = *min*⁡(*G*_*C*, *norm*_, *G*_*N*, *norm*_) ] are shaded. Because *G*_*C*, *norm*_depends only on γ_*OM*_, all curves corresponding to different *I*_*norm*_ overlap for a given pathway under C limited conditions. An enlarged view of the growth rate curves for *I*_*norm*_ = 0.01 (blue curves) is provided in Supplementary Figure 1.

Figure 5 shows the transition of microbial growth rate from C- to energy- to N-limitation under anoxic conditions when ${\text{NO}}_{3}^{-}$ is used both as EA and N_Inorg_-source *via* DNRA (solid curves) or denitrification (dashed curves) pathway. The normalized growth rate for both pathways increases linearly at low values of γ_*OM*_, attains a maximum, and then decreases non-linearly for high values of γ_*OM*_ (Figure 5A). Under C-limited conditions, the growth rate is higher for the denitrification as compared to the DNRA pathway for all values of γ_*OM*_ (cf. dashed vs. solid brown curves in Figure 5A). Conditions transition to N-limitation at high γ_*OM*_ when *I*_*norm*_ < 1.5 (colored curves). To clarify where this transition occurs, Figure 5B shows the full responses of *G*_*C, norm*_ (only C-limitation; black curves) and *G*_*N, norm*_ (only N-limitation; colored curves) to changes in γ_*OM*_. For a given level of *I*_*norm*_, the N-limited branch of the growth curve is initially flat, then decreases with increasing γ_*OM*_. This is because under N-limited conditions, CUE per unit of total ${\text{NO}}_{3}^{-}$utilized (*e*/*y*_*N*_) decreases as γ_*OM*_increases for reduced compunds (γ_*OM*_ > 4.7). Changing the level of external N_Inorg_ (*I*_*norm*_) simply re-scales the growth rate under N-limited conditions because *G*_*N*, *norm*_ = (*e*/*y*_*N*_)*I*_*norm*_ [Eq. (4)]. Under N-limitation and in contrast to C-limited conditions, the growth rate is higher for DNRA as compared to the denitrification pathway (cf. solid *vs.* dashed blue curves in Figure 5A, enlarged view provided in Supplementary Figure 1).

#### Microbial Growth on Organic Matter Containing N

**FIGURE 6:**
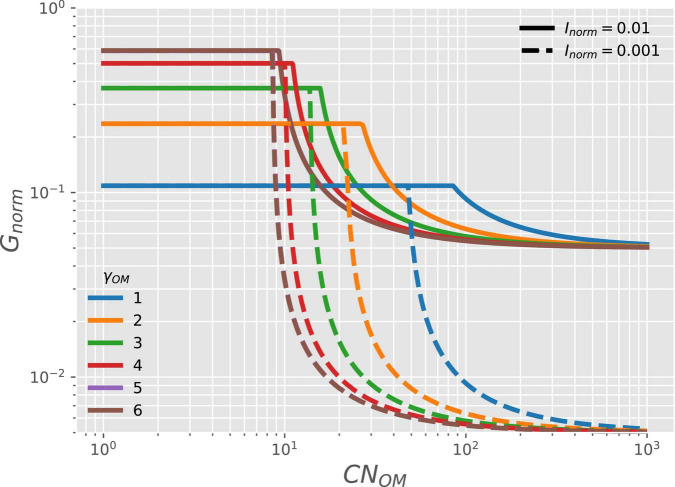
OM containing N: variation of normalized microbial growth rate (*G*_*norm*_) along a gradient of OM C:N ratio *[CN*_*OM*_ in C-mol (N-mol)^–1^] under oxic conditions, and with varying degree of reduction of the OM (γ_*OM*_; curves with different colors) and two levels of N_Inorg_-availability (*I*_*norm*_, solid *vs.* dashed curves). For γ_*OM*_ > 5, curves are close to each other so that the curve for γ_*OM*_ = 5 is not visible and is below the brown curve.

The microbial growth rate for given γ_*OM*_ (curves with different colors in Figure 6) and availability of N_Inorg_ (${\text{NH}}_{4}^{+}$; solid *vs.* dashed curves) is stable at low OM C:N ratio (*CN*_*OM*_) when organic N is abundant (C- and energy-limitations). Under these conditions, the microbial growth rate also increases with increasing γ_*OM*_ and it does not depend on N-availability (as shown in Figure 3). In contrast, under N-limited conditions, the growth rate depends on both γ_*OM*_ and *CN*_*OM*_ (Table 2). As *CN*_*OM*_ increases, the supply of organic N for microbial growth decreases, up to the point where growth becomes limited by the supply of N_Inorg_. Under these conditions, the growth rate is reduced to match the N_Inorg_ supply (Table 2); therefore, the growth rate also decreases with decreasing *I*_*norm*_ (solid *vs.* dashed curves). Contrary to the negative effect of low γ_*OM*_ under C-limitation, low γ_*OM*_ reduces the effects of N-limitation by forcing microbes to grow at a slower rate, which lowers their N-demand.

**FIGURE 7:**
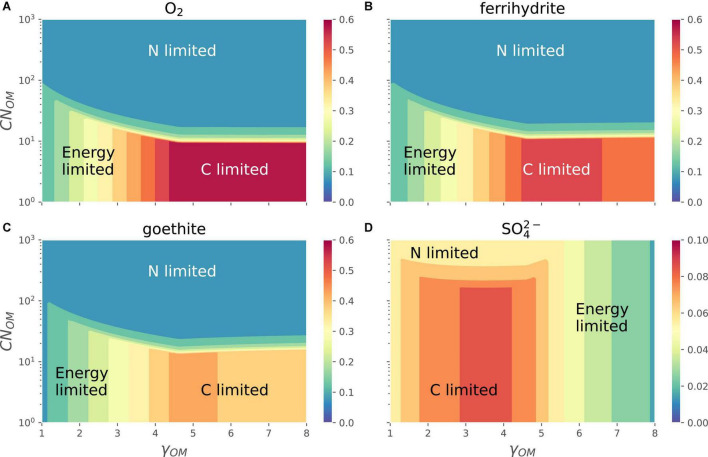
OM containing N: variation of normalized microbial growth rate (*G*_*norm*_; contours with different colors) along a gradient of OM C:N ratio *[CN*_*OM*_ in C-mol (N-mol)^–1^] and OM degree of reduction (γ_*OM*_) for different EAs [panels **(A–D)**]. A constant value of *I*_*norm*_ = 0.01 was assumed in all panels. Note that the color scale in the bottom right panel is different from those in the other three panels **(A–C)**.

The different EAs shift the relative position of energy-, C-, and N-limitation regions in the space of *CN*_*OM*_ and γ_*OM*_ (Figure 7). In the case of O_2_ and Fe^3+^ (goethite or ferrihydrite) as EAs, the growth rate varies as in Figure 6, i.e., it is maximum for OM with high γ_*OM*_ and low *CN*_*OM*_ (C-limited region), decreases with decreasing γ_*OM*_ (energy-limited region), and is lowest at high values of *CN*_*OM*_ (N-limited region). In all these cases the growth rate decreases only slightly when OM is highly reduced. In the case of ${\text{SO}}_{4}^{2-}$ as the EA (see also the orange curve in Figure 3), the growth rate at a given *CN*_*OM*_ first increases with increasing γ_*OM*_, reaches its maximum values for γ_*OM*_ close to 4 and then decreases. For lower values of γ_*OM*_, the growth rate decreases as *CN*_*OM*_ increases because of transition from C- to N-limited conditions, similar to the behavior under other EA, whereas at high γ_*OM*_ N-limitation does not occur even at *CN*_*OM*_ as high as 1,000 C-mol (N-mol)^−1^. As a result, the energy-limited region shifts from left [Figure 7, panels (A–C)] where it is caused by low γ_*OM*_, to the right in panel D where it is caused by high |Δ_*r*_*G*_*B*_| (see Supplementary Figure 2 for Δ_*r*_*G*_*B*_ as function of γ_*OM*_).

## Discussion

### Microbial Growth: Missing Link Between Bioenergetic and Stoichiometric Regulation in Biogeochemical Models

Traditionally, biogeochemical models assume that microbes are limited by C- or N-availability, depending on C:N ratio of OM and availability of N_Inorg_ (${\text{NH}}_{4}^{+}$or ${\text{NO}}_{3}^{-}$). These limitations regulate the rate of OM decomposition and the partitioning of C and N between growth and mineralized products (Cherif and Loreau, 2007; Manzoni et al., 2017). Similar stoichiometric constraints are implemented in models describing decomposition in litter and soil [e.g., Manzoni and Porporato (2009), Wutzler et al. (2017), and Zhang et al. (2018)], or in the water column or sediments of aquatic systems [e.g., Schultz and Urban (2008) and Webster et al. (2009)]. These models, except for recent developments (Song et al., 2020), tend to neglect energetic constraints on both C fluxes and their partitioning between growth and respiration, or implicitly assume that C- and energy-limitations are equivalent.

Building on previous bioenergetics theory, here we formulate microbial growth as a function of C and N stoichiometric constraints and include energy-limitation as an additional constraint acting *via*: (i) the change in Gibbs energy of OM oxidation, which is controlled by the degree of reduction of the OM (γ_*OM*_), and (ii) the change in Gibbs energy of EA reduction (Δ_*red*_*G*_*EA*_). These factors affect the efficiency of OM conversion into biomass (i.e., CUE), thus determining the microbial growth rate. These two bioenergetic factors interact with two biogeochemical ones: (iii) availability of organic or N_Inorg_ for growth (measured by *CN*_*OM*_ and *I_N_*, respectively) and (iv) availability of C for growth and catabolism (*U*_*OM*_). In natural environments, either of these four factors can become limiting and thus may reduce microbial activity. For example, when nutrients are not limiting, fluctuating soil moisture would change the redox status, forming zones of high or low microbial activity because of varying degree of the reduction potential of the local EA, even if the supply of organic C and its energetic content are spatially uniform (LaCroix et al., 2019). Notably, these four factors can interact. For instance, energy-limitation can lower N-demand, thereby reducing N-limitation, while N-limitation, when microbes metabolize oxidized N (such as ${\text{NO}}_{3}^{-}$) for growth, creates an additional energy demand on the available OM.

In the following, we discuss our results regarding (i) C- and energy-limitation (section “C- and Energy-Limitations: Effects of Organic Matter Degree of Reduction and Electron Acceptor Energy Yield on Growth”; answering question 1 in the Introduction), (ii) N- and energy-limitation (section “N- and Energy-Limitations: Effects of Different N_Inorg_-Sources on Growth” and “N- and Energy-Limitations: Using Nitrate as Both Electron Acceptor and N_Inorg_-Source on Growth”; answering question 2 in the Introduction), and (iii) combined C-, N-, and energy-limitation (section “C-, N-, and Energy-Limitations: Effects of Combined Thermodynamic and Biogeochemical Factors on Microbial Growth”; answering question 3 in the Introduction). We then conclude with a broader discussion on the limitations and implications of the proposed bioenergetic framework (section “Outlook: Approach Limitations and Implications of Bioenergetics in Microbial Ecology”).

### Interactions Among C-, N-, and Energy-Limitations

#### C- and Energy-Limitations: Effects of Organic Matter Degree of Reduction and Electron Acceptor Energy Yield on Growth

The overall patterns in normalized growth rate or CUE with the degree of reduction and type of N_Inorg_-source for microbial growth are similar to those described in other studies (Roels, 1980b; Heijnen et al., 1992; Smeaton and Van Cappellen, 2018). When microorganisms catabolize OM using high energy yielding EA such as O_2_ or ${\text{NO}}_{3}^{-}$, their growth rate increases with the increasing energy content of the OM utilized, i.e., γ_*OM*_ (Figure 3). Garayburu-Caruso et al. (2020) showed decreased respiration rates under oxic conditions for more reduced OM (high γ_*OM*_). Based on our framework, we interpreted their observations as low respiration rate coupled with high CUE (thus, high growth rate) when more reduced compounds are decomposed. However, if the catabolism is performed using low energy yielding EA (low values of |−Δ_*red*_*G*_*EA*_|), the growth rate increases with increasing γ_*OM*_ at first, but then decreases to a value close to zero for more reduced OM (Figure 3). The key to understanding this pattern is the variation of Δ_*cat*_*G*_*OM*_ and Δ_*ox*_*G*_*OM*_ with γ_*OM*_ (Supplementary Figure 2). Δ_*ox*_*G*_*OM*_ (black curve, Supplementary Figure 2) is negative for γ_*OM*_ < 1.88 and positive for γ_*OM*_ > 1.88; therefore, for γ_*OM*_ < 1.88 a low energy yielding EA would result in overall negative Δ_*cat*_*G*_*OM*_ (blue or orange curve Supplementary Figure 2A). This means that the catabolic reaction is still feasible, although with lower CUE because of the low magnitude of Δ_*cat*_*G*_*OM*_. For γ_*OM*_ > 1.88, Δ_*ox*_*G*_*OM*_ is positive, so that a low energy yielding EA could result in overall positive Δ_*cat*_*G*_*OM*_ values representing a non-spontaneous reaction, which means that the catabolic reaction is not feasible and uptake of OM stops. Such a reaction would lead to CUE = 0 in Figure 3. Moreover, for intermediate values of |−Δ_*red*_*G*_*EA*_| (e.g., EAs such as Fe^3^, ${\text{SO}}_{4}^{2-}$), the growth rate or CUE decreases with γ_*OM*_ above γ_*OM*_ > 4.7 (Figure 3) because microbes dissipate Gibbs energy (|Δ_*r*_*G*_*B*_| from the overall metabolic reaction) faster than it is produced from the catabolic reaction with increasing γ_*OM*_ (Supplementary Figure 2, panel C). However, this is not the case when O_2_ is the EA as CUE remains a monotonically increasing function of γ_*OM*_. Thus, purely C-limited conditions are only attained when the substrate is reduced and oxygen is the EA; in other conditions, the energy-limitation reduces microbial growth, even though the reaction rates still scale with substrate C content (C-energy co-limitation).

Our results show an inhibition effect of reduced OM (high γ_*OM*_) on growth when catabolism is coupled with low energy yielding EAs such as Fe^3+^ or ${\text{SO}}_{4}^{2-}$, as an outcome of decreased growth rate caused by low CUE (Figures 3, 7). Under energy-limited environments, if microbial growth is not limited by the supply of C, then our framework would predict higher respiration for more reduced compounds caused by low CUE. For example, when sulfate reducing bacteria are grown in batch or chemostat with ethanol, acetate, or lactate as substrates, more hydrogen sulfide (a proxy for respiration) was produced with ethanol compared to the other two substrates (White and Gadd, 1996). This is expected because ethanol produces more electrons compared to acetate and lactate per C mol; therefore, more ${\text{SO}}_{4}^{2-}$is used, resulting in higher respiration. Further, Zheng et al. (2019) showed that as O_2_ becomes limiting, growth is mainly controlled by CUE, and respiration remained unchanged since the supply of dissolved organic C did not change during their incubation experiment (Zheng et al., 2019). However, in some natural environments such as marine sediments or deep soil, the respiration rate of reduced OM can decrease under energy-limitation imposed by low energy yielding EA (Jin and Bethke, 2007; LaRowe et al., 2012; Boye et al., 2017; Keiluweit et al., 2017). This decrease in metabolic rates (respiration rate) is explained using a thermodynamic factor that decreases as the energy produced from catabolism decreases. Such natural environments are often limited in the availability of C as well, so the microbial metabolism is restricted to maintenance functions (or basal power requirement). In other words, microbes are under a physiological survival state without significant growth, while in our framework, we focus on respiration processes that are coupled to growth.

Furthermore, Worrall et al. (2018) showed that the Gibbs energy of formation of particulate OM decreases with depth in peatlands, which implies that the Gibbs energy of combustion (assuming O_2_ as EA) increases with depth. Worrall et al. (2018) related this observation to the accumulation of OM in peatlands, as OM becomes “thermodynamically inhibited” for microbial uptake and can thus remain in the system. In Figure 3, we showed a similar effect. With increasing depth, oxygen-availability decreases, other EAs become available, and when OM oxidation is coupled with low energy yielding EAs, Gibbs energy produced from catabolism decreases even if OM is composed of labile C such as glucose. As a result of energy-limitation, growth rate and CUE decrease with depth. Therefore, our approach provides an alternative explanation to Worrall et al. (2018) results, in which the thermodynamic factor proposed by Jin and Bethke (2007) was used to explain thermodynamic inhibition.

Understanding the interplay between C- and energy-limitations requires considering all components of bioenergetic regulation of OM decomposition, because the degree of reduction of OM (and thus its chemical nature) controls decomposition together with the coupled half-reaction of the EA reduction. The latter might constrain microbial growth even on energy-rich OM.

#### N- and Energy-Limitations: Effects of Different N_Inorg_-Sources on Growth

The metabolism of ${\text{NO}}_{3}^{-}$ varies across microorganisms and with environmental conditions (Lin and Stewart, 1997; Kraft et al., 2014; Kuypers et al., 2018). For example, CUE and growth rate are decreased if ${\text{NO}}_{3}^{-}$ is used instead of ${\text{NH}}_{4}^{+}$ as the sole N-source (Stouthamer, 1977; Wray et al., 1996) (Figure 4). Our model explains this decrease with the energetic cost of ${\text{NO}}_{3}^{-}$ reduction, providing a complementary explanation to the inhibition of ${\text{NO}}_{3}^{-}$-uptake in the presence of ${\text{NH}}_{4}^{+}($Kobayashi and Ishimoto, 1973; Rice and Tiedje, 1989; Polcyn and Luciński, 2003). Moreover, the transition from N- to energy-limitation (or *vice versa*) depends on different processes under oxic and anoxic conditions. Under oxic conditions, the energy-limitation caused by a low degree of reduction of the OM alleviates N-limitation when N-availability decreases. In fact, the transition from energy to N-limitation occurs at lower N-availability when γ_*OM*_ decreases, because N-demand at low γ_*OM*_ is also lower (Figures 4, 6). Thus, under oxic conditions, shifts in N-demand driven by energy availability define the transition from energy- to N-limitation.

#### N- and Energy-Limitations: Using Nitrate as Both Electron Acceptor and N_Inorg_-Source on Growth

Under anoxic conditions, when ${\text{NO}}_{3}^{-}$ is used as both EA and N_Inorg_-source for biomass, microorganisms compete for ${\text{NO}}_{3}^{-}$ reduction *via* denitrification or DNRA pathway. As a result, the N metabolic pathway (e.g., denitrification *vs.* DNRA), by determining the N-demand for catabolic and anabolic processes, controls how the transition between energy- and N-limitations occurs (Figure 5).

On the one hand, denitrification may cause N-limitation by removing N from the system, whereas DNRA simply reduces ${\text{NO}}_{3}^{-}$ to ${\text{NH}}_{4}^{+}$. In fact, under N-limited conditions, denitrifiers need to reduce an additional amount of ${\text{NO}}_{3}^{-}$ to ${\text{NH}}_{4}^{+}$ to meet their N-demand for growth compared to the DNRA pathway, which produces excess ${\text{NH}}_{4}^{+}$ through catabolism. Therefore, when ${\text{NO}}_{3}^{-}$-availability is low, microbes performing DNRA would outcompete denitrifiers, as DNRA allows higher growth rate than denitrification for all values of γ_*OM*_ (Figure 5B, solid *vs.* dashed curves). On the other hand, denitrification produces more Gibbs energy per electron transferred to oxidation of OM compared to DNRA, which results in overall a higher Gibbs energy of catabolism for 1 C-mol of OM (Supplementary Table 1). Therefore, with abundant ${\text{NO}}_{3}^{-}$, microbial CUE for denitrification is higher than for the DNRA pathway (Figure 5B, dashed *vs.* solid black curves). As a result, microbial N-demand is always met, and growth is limited by the energy produced from catabolism; hence, denitrifiers have a higher growth rate than microbes performing DNRA for all values of γ_*OM*_ (Figure 5A, dashed *vs.* solid curves for *I*_*norm*_ = 1.5).

Between these two extreme cases of ${\text{NO}}_{3}^{-}$poor *vs.* ${\text{NO}}_{3}^{-}$ rich conditions, the microbial growth rate of two pathways varies depending on γ_*OM*_ (Figure 5). At low values of γ_*OM*_, growth is energy-limited and since denitrification produces more Gibbs energy, denitrifiers are likely dominant. In contrast, at high values of γ_*OM*_, N-limitation becomes the controlling factor, so that microbes performing DNRA would dominate. With labile OM, such as glucose or acetate (both with degree of reduction 4), our theory would predict dominance of the DNRA pathway, because of higher growth rate at low nitrate concentration compared to dominance of denitrification pathway at high nitrate concentration (see Figure 5A). This prediction is similar to observations from field, lab or modeling studies (Kraft et al., 2014; van den Berg et al., 2016; Putz et al., 2018). However, most experimental studies ignore the role of OM degree of reduction as C is provided in labile form (e.g., glucose or acetate), and N_Inorg_-availability is manipulated by adding ${\text{NO}}_{3}^{-}$ or ${\text{NH}}_{4}^{+}$ (van den Berg et al., 2016; Putz et al., 2018). Therefore, our results from Figures 4, 5 could be used to generate hypotheses or explain empirical results where the dominance of microbial communities is assessed by altering the quality of added substrates (its degree of reduction) under varying N_Inorg_-availability. For example, we predicted higher denitrifier growth rate feeding on oxidized OM, but higher growth rate of microbes performing DNRA feeding on reduced OM under moderate nitrate-availability (Figure 5A, green lines).

#### C-, N-, and Energy-Limitations: Effects of Combined Thermodynamic and Biogeochemical Factors on Microbial Growth

When all three limitations—C, N, and energy—are considered, the overall patterns in microbial growth rate remain similar to those described in previous sections. Under oxic conditions, the effects of N-limitation on growth rate are decoupled from those arising under C- and energy-limitations (Figures 6, 7A). Under C-limitation, the growth rate is mainly determined by the CUE, which is controlled by γ_*OM*_, whereas under N-limitation, the growth rate is determined by the N-imbalance, which is controlled by the C:N ratio of OM and N_Inorg_-availability. The transition point between C- and N-limitations occurs at progressively higher N-availability as the N-demand increases with more reduced OM. Garayburu-Caruso et al. (2020) showed that observed respiration rates under C excess (high concentration of C-, N-limited) conditions were controlled by N-availability, whereas under C-limited (low concentration of C) conditions, they were controlled by the degree of reduction of OM, indicating bioenergetic regulation of OM decomposition only under C-limitation; however, this is only valid for oxic conditions. When the growth rate is analyzed in the *CN*_*OM*_−-γ_*OM*_ space, contrasting patterns emerged with the type of EA utilized [compare panels (A) and (D) in Figure 7]. For example, the dominating factors, C-, N-, or energy-limitation, controlling the growth rates are switched as the energy content of OM increases (γ_*OM*_). This is explained by noting that when catabolism is coupled with low energy yielding EAs, the overall energy obtained from catabolism is very small; therefore, decreasing the growth rate as CUE is decreased (Figure 3).

#### The Case of Overflow Respiration Response Under N-Limited Conditions

We tested how energy-limitation would affect the growth rate, if microbes were to respire more rather than reducing OM-uptake during N-limitation. In fact, under N-limited conditions, the growth rate defined by Eq. (6) assumes that the growth is reduced by decreasing OM-uptake rate through the stoichiometric coefficients for N (Table 2), similar to the “N inhibition” mechanism described by Manzoni and Porporato (2009). This mechanism represents a downregulation of extracellular enzyme production. However, other strategies for microbial growth under N-limitation can have a different effect on the growth rate (Manzoni et al., 2021). One putative mechanism is overflow respiration or exudation of excess C, which suggests that under N-limitation, microbes do not reduce the uptake of OM; instead, they remove extra C by overflow respiration (Sistla et al., 2012; Wild et al., 2014; Wutzler et al., 2017), higher investment in extracellular enzymes, or possibly exuding more. This is mathematically achieved by reducing microbial CUE at a constant uptake rate of OM (Manzoni et al., 2017). We tested whether this mechanism would affect growth rate when varying γ_*OM*_, *CN*_*OM*_, and availability of different EAs in Supplementary Figures 3, 4. As expected, the variation of growth rate with γ_*OM*_ under C-limited conditions remains the same as shown in Figures 6, 7. However, under N-limitation, the growth rate is much higher when overflow respiration is performed, because the supply of N from the OM is not lowered (compare Supplementary Figure 3 *vs.* Figure 6, and Supplementary Figure 4 *vs.* Figure 7). This metabolic regulation would thus appear to be “optimal” (Manzoni et al., 2017) because it allows higher growth rate compared to reducing the substrate uptake rate. As *CN*_*OM*_ increases under N-limitation, the CUE itself decreases, so that all growth curves converge to a single curve for a given N_Inorg_-availability and regardless of γ_*OM*_.

### Outlook: Approach Limitations and Implications of Bioenergetics in Microbial Ecology

While we studied microbial growth for given environmental conditions, in natural settings, amount and quality of OM, availability of oxygen and inorganic EAs, and microbial biomass stoichiometry and community composition vary through time and at different time scales. Applying bioenergetics in a dynamic context where both state variables (mass and energy of substrates and microbial compartments) and environmental conditions change through time would require complete mass and energy balance equations, including the definition of the rates of consumption and transport of mass and energy. For example, dynamic simulations show that DNRA and denitrification pathways coexist for a range of C:N ratio of OM and oxic/anoxic conditions (van de Leemput et al., 2011; van den Berg et al., 2016; Jia et al., 2020; Zakem et al., 2020); however, our study could not capture such behavior because our formulation is time implicit.

Moreover, in a dynamic system, the degree of reduction of degrading OM changes during decomposition, which provides a bioenergetic link to the continuous nature of chemical changes and OM stability (Williams and Plante, 2018). Systems with fluctuating redox status may face frequent changes from energy-rich to energy-limited conditions; therefore, more reduced OM can accordingly become energetically favorable or unfavorable, and C storage would depend on the time scale of such fluctuations (Santruckova et al., 2005; Bhattacharyya et al., 2018; LaCroix et al., 2019; Lin et al., 2021). Similarly, in energy-limited systems (anoxic conditions with lower availability of favorable EA), more reduced compounds are energetically unfavorable, which may lead to their longer turnover time, and thus accumulation (Boye et al., 2017; Keiluweit et al., 2017).

We also assumed standard conditions to simplify Gibbs energy calculations, whereas in natural systems the concentrations of reaction species change through time. Therefore, Gibbs energy calculations of catabolic and anabolic reactions, and the associated stoichiometric coefficients, would need to be time-dependent. Further, we assumed that microorganisms adapt to N-limitation based on their fixed elemental ratio, which has been reported to be one of the limitations of Liebig’s law of the minimum (Tang and Riley, 2021). Allowing microorganisms to change their elemental ratio (i.e., *CN*_*B*_) to balance resource acquisition would affect CUE as well as other stoichiometric coefficients [e.g., Eqs. (28) and (29)]. Flexible microbial C:N could also have long-term consequences in dynamic contexts such as during litter decomposition (Manzoni et al., 2021)—higher C:N could allow higher allocation of C to growth, resulting in more necromass that could be ultimately stabilized in soil. Thus, our formulation should be expanded to be used under non-standard and dynamic conditions.

Despite the limitations of our approach and the complexities inherent in coupling mass and energy balances, bioenergetic approaches are promising to explain patterns in microbial growth rate (Helton et al., 2015; Calabrese et al., 2021) and microbial community structure (Großkopf and Soyer, 2016; Seto and Iwasa, 2020; González-Cabaleiro et al., 2021). For example, Großkopf and Soyer (2016) showed how two microbial species can coexist at a steady state using a coupled kinetic and bioenergetic growth model under energy-limited conditions. Traditional kinetic models (Monod equation) could not predict such behavior. Furthermore, bioenergetics-based models provide tools to link genome to population scale models [Shapiro et al. (2018) and Ref. therein, Dukovski et al. (2021)]. New models are exploring the potential of bioenergetics to study how microbial metabolic diversity and spatial heterogeneity of resources interact and shape community dynamics and resource niches in the complex soil environment (Araujo Granda et al., 2016; Jayathilake et al., 2017; Borer et al., 2019; Gogulancea et al., 2019; Li et al., 2019; Ben Said et al., 2020; Calabrese et al., 2020; Dal Co et al., 2020; Desmond-Le Quéméner et al., 2021). Bioenergetics can thus be a useful complement to traditional biogeochemical models describing only the dynamics of C and nutrients.

## Conclusion

The bioenergetic principles provide a unified theory for integrating kinetic and stoichiometric constraints on microbial growth. In this work, we showed how bioenergetics could be used to link the stoichiometry of microbial growth under different types of metabolisms and varying environmental conditions. Specifically, we quantified microbial growth rate in C-, N-, and energy-limited systems and used our theory to explain patterns in growth rate using two bioenergetic variables, i.e., the degree of reduction of the OM (γ_*OM*_) and the energy yield of the EA reduction, and two biogeochemical ones, i.e., availability of organic N or N_Inorg_ for growth (*CN*_*OM*_, *I*_*norm*_) and of C for growth and catabolism (*U*_*OM*_). In general, under C-rich and energy-limited conditions, the growth rate peaks at intermediate γ_*OM*_ and increases with high energy-yielding EA. Our analysis also qualitatively explains microbial activity patterns across a range of metabolic pathways (aerobic, denitrification, DNRA). We showed that energy-limitations could reduce N-limitation by decreasing CUE, and N-limitation exacerbates energy-limitation by imposing additional energy requirements such as nitrate reduction in denitrification. Applications of bioenergetics provide a powerful tool that can be used to study microbial growth dynamics and diverse metabolic pathways. Since metabolic diversity is closely related to microbial community diversity, bioenergetics could prove valuable to understand patterns in microbial ecology driven by gradients of energy- and nutrient-availabilities.

## Data Availability Statement

The original contributions presented in the study are included in the article/Supplementary Material, further inquiries can be directed to the corresponding author.

## Author Contributions

AC designed the study and developed the theory with feedback from SM and SC and implemented the framework, produced the results, and drafted the manuscript. All authors commented and revised the manuscript.

## Conflict of Interest

The authors declare that the research was conducted in the absence of any commercial or financial relationships that could be construed as a potential conflict of interest.

## Publisher’s Note

All claims expressed in this article are solely those of the authors and do not necessarily represent those of their affiliated organizations, or those of the publisher, the editors and the reviewers. Any product that may be evaluated in this article, or claim that may be made by its manufacturer, is not guaranteed or endorsed by the publisher.
