# Supplementary material for: Interacting Bioenergetic and Stoichiometric Controls on Microbial Growth
Source: Front Microbiol. 2022 May 17;13:859063. doi: 10.3389/fmicb.2022.859063 (PMC9152356; doi:10.3389/fmicb.2022.859063)
Supplement: Supplementary file 1 [file Data_Sheet_1.pdf]

Supplementary information

Interacting bioenergetic and stoichiometric controls on microbial growth

Arjun Chakrawal[1, 2*], Salvatore Calabrese[3], Anke M. Herrmann[4], and Stefano Manzoni[1, 2]

[1]Department of Physical Geography, Stockholm University, 10691 Stockholm, Sweden

[2]Bolin Centre for Climate Research, Stockholm University, 10691 Stockholm, Sweden

[3]Department of Biological and Agricultural Engineering, Texas A&M University, 333 Spence St, College Station, TX 77843 USA.

[4]Department of Soil and Environment, Swedish University of Agricultural Sciences, Uppsala, 750 07, Sweden

* Correspondence:
Arjun Chakrawal
arjun.chakrawal@natgeo.su.se

1 Supplementary Tables

Table S1. Half-reactions of reduction of electron acceptors (EAs), moles of electron received by the EA (γ_{EA}), and change in Gibbs energy ($\Delta_{red}G_{EA}$) expressed per mol of EA or per mol of electron (LaRowe and Amend, 2015; Dick, 2019).

Compound	γ_{EA}	Half-reaction	$\Delta_{red}G_{EA}$ (kJ/mol EA)	$\frac{\Delta_{red}G_{EA}}{\gamma_{EA}}$ (kJ/mol e^-)
O_2 (oxygen)	4	$O_2 + 4e^- + 4H^+ \rightarrow 2H_2O$	-490.9	-122.72
Mn^{+4}(pyrolusite)	2	$MnO_2 + 4H^+ + 2e^- \rightarrow Mn^{2+} + 2H_2O$	-240	-120
Fe^{+3} (goethite)	1	$FeOOH + 3H^+ + e^- \rightarrow Fe^{2+} + 2H_2O$	-75.6	-75.6
Fe^{+3} (ferrihydrite)	1	$FeOOH + 3H^+ + e^- \rightarrow Fe^{2+} + 2H_2O$	-100.6	-100.6
$SO4^{-2}$ (sulfate)	8	$SO4^{2-} + 8e^- + 9H^+ \rightarrow HS^- + 4H_2O$	-192.3	-24.04
			$\Delta_{red}G_{EA} = \Delta_{red}G_N$ (kJ/mol NO_3^-)	
NO_3^- (nitrate, DNRA)	8	$NO_3^- + 8e^- + 10H^+ \rightarrow NH_4^+ + 3H_2O$	-680	-85
NO_3^- (nitrate, denitrification)	8	$NO_3^- + 5e^- + 6H^+ \rightarrow 0.5\,N_2 + 3H_2O$	-591.5	-118.2

Table S2. Example of reaction for microbial growth on glucose/oxygen/nitrate and glycine/nitrate/nitrate as organic matter (OM)/electron acceptor (EA)/ inorganic N source, including chemical equations and change in Gibbs energy (ΔG).

Reaction	Chemical equation (Glycine/NO_3^-/ NO_3^-)	ΔG
Catabolism	OM oxidation: $CH_{2.5}ON_{0.5} + 2H_2O \rightarrow HCO_3^- + 0.5NH_4^+ + 3.5\,H^+ + 3e^-$	48.9 kJ (C-mol glycine)$^{-1}$
	EA reduction: $NO_3^- + 8e^- + 10H^+ \rightarrow NH_4^+ + 3H_2O$	-680 kJ (mol NO_3^-)$^{-1}$
	Overall catabolic reaction: $CH_{2.5}ON_{0.5} + 0.375NO_3^- + 0.875H_2O + 0.25\,H^+ \rightarrow HCO_3^- + 0.875\,NH_4^+$	-223 kJ (C-mol glycine)$^{-1}$
Anabolism	$1.4\,CH_{2.5}ON_{0.5} + 0.1H^+ + 0.3H_2O \rightarrow CH_{1.8}O_{0.5}N_{0.2} + 0.4\,HCO_3^- + 0.5NH_4^+$	-8.95 kJ (C-mol biomass)$^{-1}$
Overall metabolic reaction*	$CH_{2.5}ON_{0.5} + 0.2175\,NO_3^- + 0.6H_2O + 0.175H^+ \rightarrow 0.3\,CH_{1.8}O_{0.5}N_{0.2} + 0.6575\,NH_4^+ + 0.7\,HCO_3^-$	-137 kJ (C-mol glycine)$^{-1}$
	$3.33CH_{2.5}ON_{0.5} + 0.725\,NO_3^- + 2H_2O + 0.5833H^+ \rightarrow CH_{1.8}O_{0.5}N_{0.2} + 2.2NH_4^+ + 2.33HCO_3^-$	-465 kJ (C-mol biomass)$^{-1}$

**Reaction**	**Chemical equation (Glucose/O_2/ NO_3^-)**	ΔG
**Catabolism**	OM oxidation: $CH_2O + 2H_2O \rightarrow HCO_3^- + 5H^+ + 4e^-$	60.3 kJ (C-mol glucose)$^{-1}$
	EA reduction: $O_2 + 4e^- + 4H^+ \rightarrow 2H_2O$	-490.9 kJ (mol O_2)$^{-1}$
	N source reduction: $NO_3^- + 8e^- + 10H^+ \rightarrow NH4^+ + 3H_2O$	-680 kJ (mol NO_3^-)$^{-1}$
	Overall catabolic reaction: $CH_2O + 0.185NO_3^- + 0.63O_2 + 0.185H_2O \rightarrow 0.63\ H^+ + HCO_3^- + 0.185\ NH_4^+$	-375 kJ (C-mol glucose)$^{-1}$
**Anabolism**	$1.05\ CH_2O + 0.2\ NH_4^+ \rightarrow CH_{1.8}O_{0.5}N_{0.2} + 0.05\ HCO_3^- + 0.25\ H^+ + 0.4H_2O$	-8.95 kJ (C-mol biomass)$^{-1}$
**Overall metabolic reaction***	$CH_2O + 0.094\ NO_3^- + 0.32O_2 \rightarrow 0.47\ CH_{1.8}O_{0.5}N_{0.2} + 0.53\ HCO_3^- + 0.436H^+ + 0.097H_2O$	-192 kJ (C-mol glucose)$^{-1}$
	$2.12CH_2O + 0.2\ NO_3^- + 0.68O_2 \rightarrow CH_{1.8}O_{0.5}N_{0.2} + 1.12HCO_3^- + 0.927H^+ + 0.2\ H_2O$	-408.5 kJ (C-mol biomass)$^{-1}$

* To obtain the overall metabolic reaction for glycine and glucose, the growth efficiencies ($e_{glycine} = 0.3$ and $e_{glucose} = 0.47$) are estimated from Eq. (34) and then used Eq.(22).

Table S3. Values of Gibbs energy of formation for selected compounds at pH 7 from Kleerebezem and Van Loosdrecht (2010).

**Compound**	**Gibbs energy of formation $\Delta_f G^0$ (kJ/mol)**
Water	-237.2
Bicarbonate	-586.85
Glucose	-917.22
H^+	0
Oxygen	16.4
Nitrate	-111
Ammonium	-79.5

2 Supplementary Figures

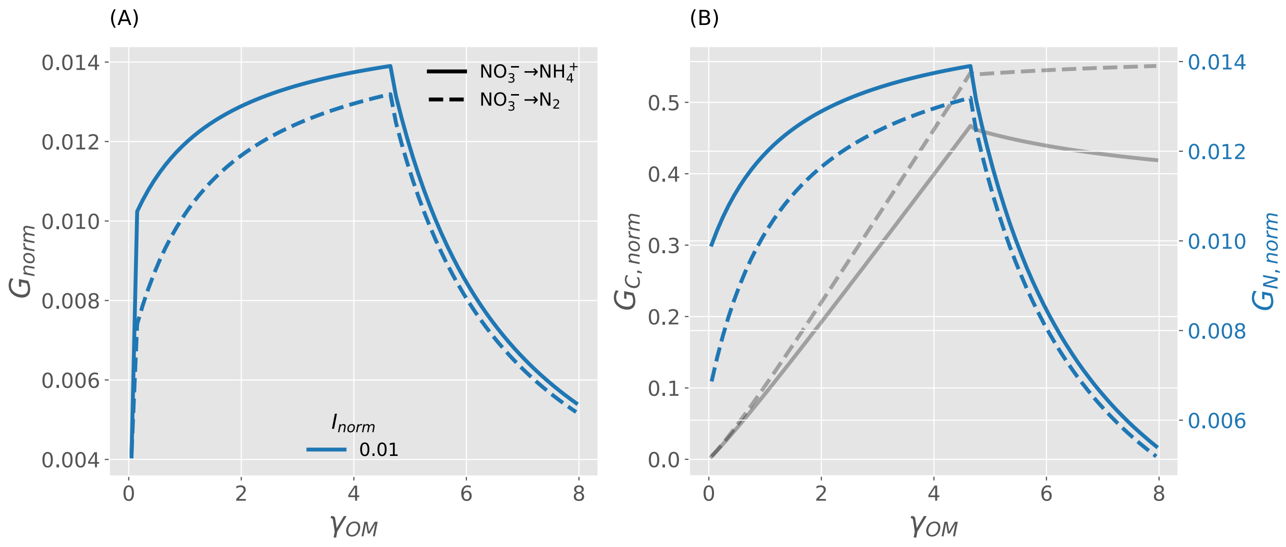

Figure S1. Enlarged view of growth rates in Figure 4 for $I_{norm} = 0.01$. (A) normalized microbial growth rate (G_{norm}) as a function of degree of reduction of the OM, when the OM is catabolized via denitrification (dashed curves) or DNRA pathway (solid curves). (B) normalized microbial growth rate under C ($G_{C,norm}$; Eq. (3)) and N ($G_{N,norm}$; Eq. (4)) limited conditions.

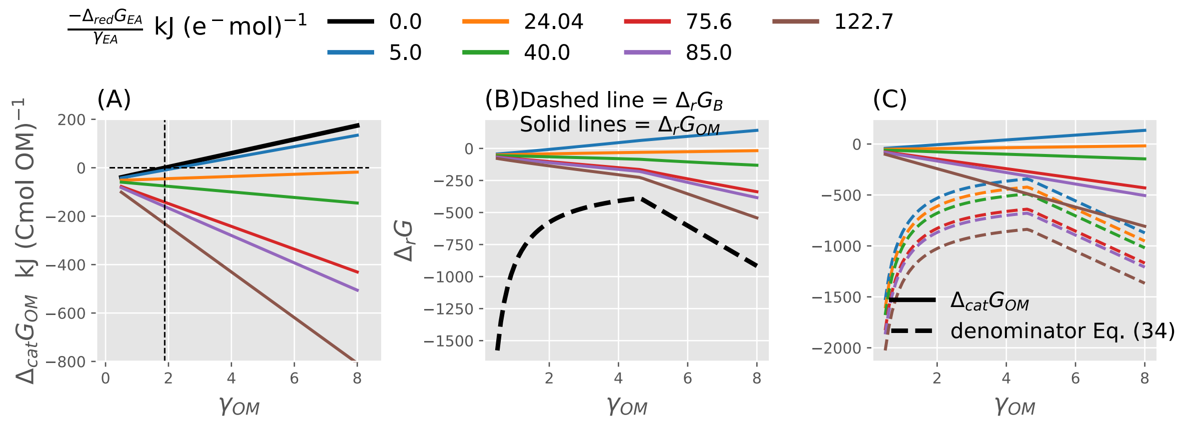

Figure S2. (A) Change in Gibbs energy dissipated from the catabolic reaction ($\Delta_{cat}G_{OM}$; kJ (C-mol OM)$^{-1}$) as a function of the degree of reduction of OM (γ_{OM}), for different values of change in Gibbs energy of the EA reduction reaction ($\Delta_{red}G_{EA}/\gamma_{EA}$; kJ per mol of electrons accepted by 1 mol of EA), shown as curves of different colors. The black line indicates $\Delta_{cat}G_{OM} = \Delta_{ox}G_{OM}$, when $\Delta_{red}G_{EA} = 0$. The thin dashed lines indicate $\Delta_{cat}G_{OM} = 0$ and $\gamma_{ED} = 1.8$, above which $\Delta_{cat}G_{OM} > 0$. (B) Change in Gibbs energy dissipated from overall reaction $\Delta_r G_{OM}$ (solid lines; kJ (C-mol OM)$^{-1}$) and $\Delta_r G_B$ (dashed line; kJ (C-mol B)$^{-1}$) as a function of γ_{ED}. (C) Numerator ($\Delta_{cat}G_{ED}$) and denominator of Eq. (34) ($\Delta_r G_B - \Delta_{ana}G_B + \gamma_B/\gamma_{OM}\,\Delta_{cat}G_{OM}$) as a function of γ_{OM}; these two terms determine trends in e with γ_{OM}.

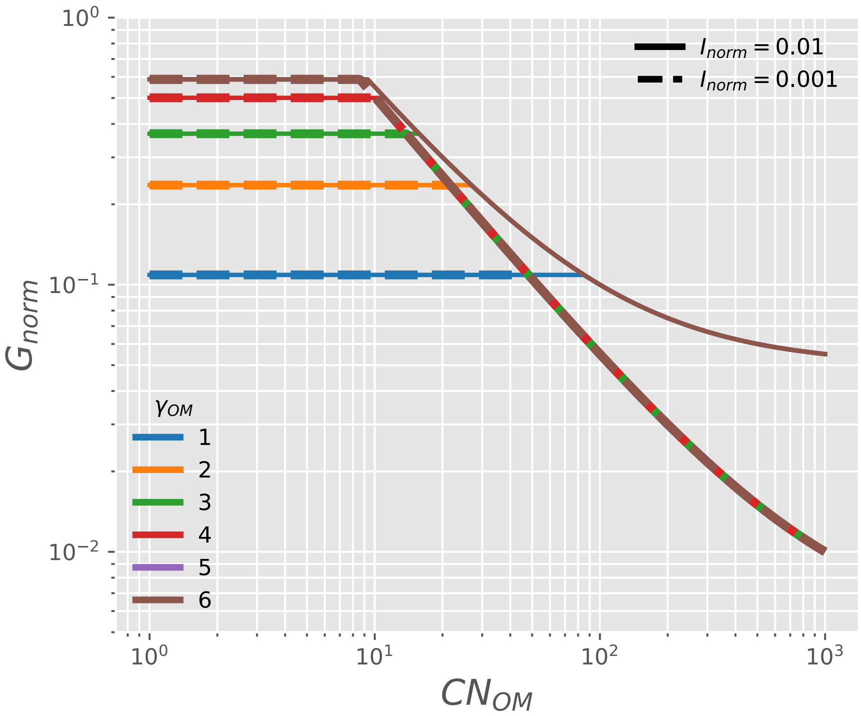

Figure S3. Variation of normalized microbial growth rate (G_{norm}) when overflow respiration is assumed instead of decreased organic matter(OM) uptake under N limitation (see Figure 5 for the latter scenario). Variations in G_{norm} are assessed along a gradient of OM C:N ratio (CN_{OM}) under aerobic conditions, and with varying degree of reduction of OM (γ_{OM}; curves with different colors), and different inorganic N availability (I_{norm}, solid vs. dashed curves).

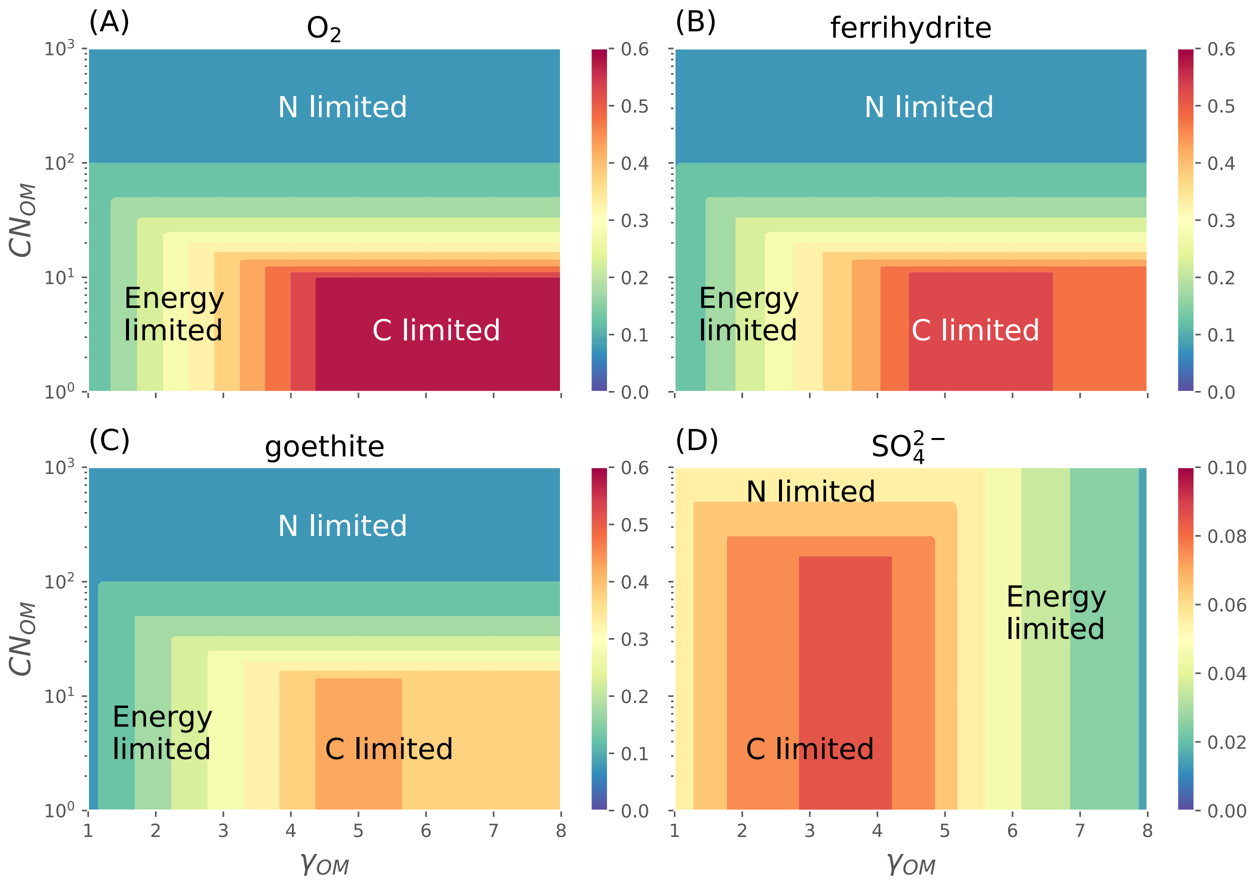

Figure S4. Variation of normalized microbial growth rate (G_{norm}; contours curves with different colors) when overflow respiration is assumed instead of decreased organic matter(OM) uptake under N limitation (see Figure 6 for the latter scenario). Variations in G_{norm} are assessed along a gradient of OM C:N ratio (CN_{OM}) and degree of reduction (γ_{OM}), for different electron acceptors. A constant value of $I_{norm} = 0.01$ was assumed.

3 Code availability

A python script used to generate all figures is provided in this Github repository https://github.com/ArjunChakrawal/Stoichiometry-and-Thermodynamics.git.
